# Low expression of ZFP36L1 in osteosarcoma promotes lung metastasis by inhibiting the SDC4-TGF-β signaling feedback loop

**DOI:** 10.1038/s41388-023-02880-7

**Published:** 2023-11-07

**Authors:** Mengjun Ma, Jiahao Zhuang, Hongyu Li, Rujia Mi, Yihui Song, Wen Yang, Yixuan Lu, Xin Shen, Yanfeng Wu, Huiyong Shen

**Affiliations:** 1 Department of Orthopedics, The Eighth Affiliated Hospital of Sun Yat-sen University, Shenzhen, 518000 China; 2 Center for Biotherapy, The Eighth Affiliated Hospital of Sun Yat-sen University, Shenzhen, 518000 China

**Keywords:** Bone cancer, Metastasis

## Abstract

ZFP36L1, which is a negative regulator of gene transcripts, has been proven to regulate the progression of several carcinomas. However, its role in sarcoma remains unknown. Here, by using data analyses and in vivo experiments, we found that ZFP36L1 inhibited the lung metastasis of osteosarcoma (OS). Knockdown of ZFP36L1 promoted OS cell migration by activating TGF-β signaling and increasing SDC4 expression. Intriguingly, we observed a positive feedback loop between SDC4 and TGF-β signaling. SDC4 protected TGFBR3 from matrix metalloproteinase (MMP)-mediated cleavage and therefore relieved the inhibition of TGF-β signaling by soluble TGFBR3, while TGF-β signaling positively regulated SDC4 transcription. We also proved that ZFP36L1 regulated SDC4 mRNA decay through adenylate-uridylate (AU)–rich elements (AREs) in its 3’UTR. Furthermore, treatment with SB431542 (a TGF-β receptor kinase inhibitor) and MK2 inhibitor III (a MAPKAPK2 inhibitor that increases the ability of ZFP36L1 to degrade mRNA) dramatically inhibited OS lung metastasis, suggesting a promising therapeutic approach for the treatment of OS lung metastasis.

## Introduction

Osteosarcoma (OS) is the main primary malignant bone tumor, and it is associated with a high risk of metastasis in young adults and children [1]. The main challenge in OS treatment is lung metastasis [2, 3]. With the help of chemotherapy, the 5-year survival rate of OS has increased to approximately 70% since the 1970s, but the 5-year survival rate remains as low as 20–30% in patients with lung metastasis [4]. However, the biological mechanism that explains why OS is highly prone to lung metastasis is still unclear.

ZFP36L1 is an adenylate-uridylate (AU)-rich RNA-binding protein [5]. ZFP36L1 contains highly conserved tandem zinc-fingers that recognize adenylate-uridylate (AU)–rich elements (AREs) in the 3’UTRs of RNA. Furthermore, ZFP36L1 interacts with RNA degradation complexes to participate in ARE-mediated RNA decay. Many studies have shown that ZFP36L1 suppresses tumor progression [6, 7]. For example, ZFP36L1 decreases numerous oncogenic transcripts that are involved in the cell cycle and hypoxic signaling pathways in bladder cancer, leading to inhibition of hypoxic adaptation, metabolism, angiogenesis, and cell cycle progression [8]. ZFP36L1 might be a powerful posttranscriptional regulator of cancer. However, whether ZFP36L1 participates in the regulation of OS progression remains unknown.

TGFBR3 is a TGF-β receptor without a functional kinase domain [9]. It promotes TGF-β signaling by sequestering and presenting ligands to TGFBR2 [10]. However, after cleavage by sheddases, the soluble form of TGFBR3 inhibits TGF-β signaling by sequestering TGF-β ligands extracellularly [11]. This mechanism renders TGFBR3 a key regulator of TGF-β signaling. However, little is known about the mechanism underlying TGFBR3 cleavage. The specific sheddases and regulators of TGFBR3 cleavage warrant further studies.

In our study, we found that ZFP36L1 was expressed at low levels in OS lung metastases. Overexpression of ZFP36L1 decreased the migration and lung metastasis of OS. We also showed that ZFP36L1 increased SDC4 mRNA decay in an ARE-mediated manner. SDC4 regulated TGFBR3 cleavage, and it inhibited TGFBR3 cleavage and therefore maintained TGF-β signaling pathway activation. Overexpression of ZFP36L1 significantly reduced SDC4 expression and SDC4-mediated TGF-β signaling pathway activation. This study elucidates the mechanism by which ZFP36L1 is involved in the lung metastasis of OS, highlighting a promising approach for ZFP36L1-targeted therapy in OS treatment.

## Results

### ZFP36L1 expression correlated with lung metastasis of osteosarcoma

**Fig. 1:**
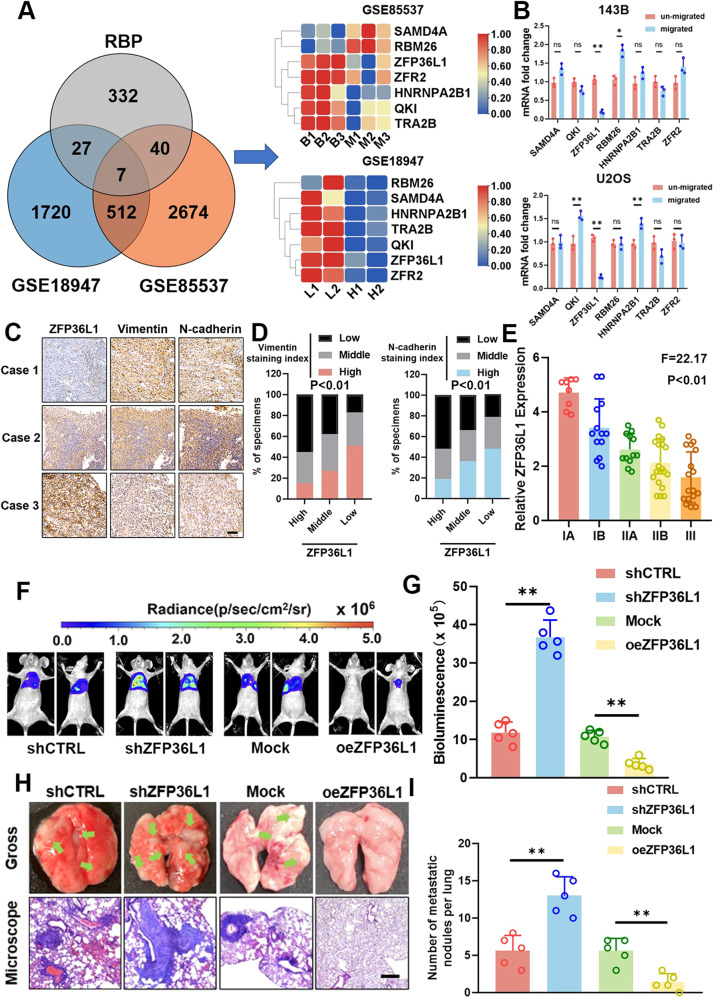
Downregulation of ZFP36L1 is correlated with the malignant progression of osteosarcoma. **A** Venn diagram showing that the expression of 7 RBPs significantly differed in GSE85537 (bone lesions vs. lung metastases) and GSE18947 (cells with low metastatic potential vs. cells with high metastatic potential). **B** q-PCR assays of RBP-encoding genes in cells that migrated or did not migrate in Transwell assays. **C** Representative IHC images of ZFP36L1, Vimentin and N-cadherin in individual samples from among 70 OS specimens. Scale bar, 100 μm. **D** The percentage of samples with low or high ZFP36L1 expression compared to the expression of Vimentin and N-cadherin. **E** Relative expression of ZFP36L1 in samples from individual OS patients with different tumor stages. Bioluminescence images (**F**) were captured to compare tumor growth in the lungs, and the bioluminescence levels (**G**) were measured by LivingImage software. **H**, **I** Representative HE staining and gross images (**H**) of lungs in mice injected with 143B cells expressing different levels of ZFP36L1. The number of lung tumors (**I**) in different groups of mice was counted. Scale bar, 120 μm. The data are shown as means ± SEMs; *P < 0.05, **P < 0.01.

RNA-binding proteins (RBPs) play an important role in cancer progression [12, 13]. Here, by analyzing RBP expression in two published datasets (GSE18947 and GSE85537), we found that 7 RBPs exhibited differential RNA expression in an OS cell subline with high metastatic potential or in OS lung metastasis samples (Fig. 1A). To further explore the role of these 7 RBPs in OS metastasis, we performed a Transwell migration assay and collected the migrated cells (below the membrane) and unmigrated cells (above the membrane). The q-PCR results showed that ZFP36L1 expression was significantly decreased in the migrated cells (Fig. 1B). We also performed immunohistochemistry (IHC) staining of ZFP36L1, Vimentin and N-cadherin in 70 OS samples. Statistically significant negative correlations between ZFP36L1 and Vimentin expression and between ZFP36L1 and N-cadherin expression were observed (Fig. 1C, D). We also found that ZFP36L1 expression was lower in samples with a high Enneking stage (Fig. 1E). These results showed that ZFP36L1 expression correlates with the metastasis of OS.

Furthermore, we constructed ZFP36L1-knockdown (shZFP36L1) and overexpression (oeZFP36L1) 143B cell lines (Fig. S1) and performed an in vivo lung metastasis assay. The results showed that shZFP36L1 143B cells markedly increased tumor colonization of the lung, while oeZFP36L1 143B cells reduced tumor colonization (Fig. 1F–I). These results suggested that ZFP36L1 inhibited the lung metastasis of OS.

### ZFP36L1 inhibited OS migration through EMT regulation

**Fig. 2:**
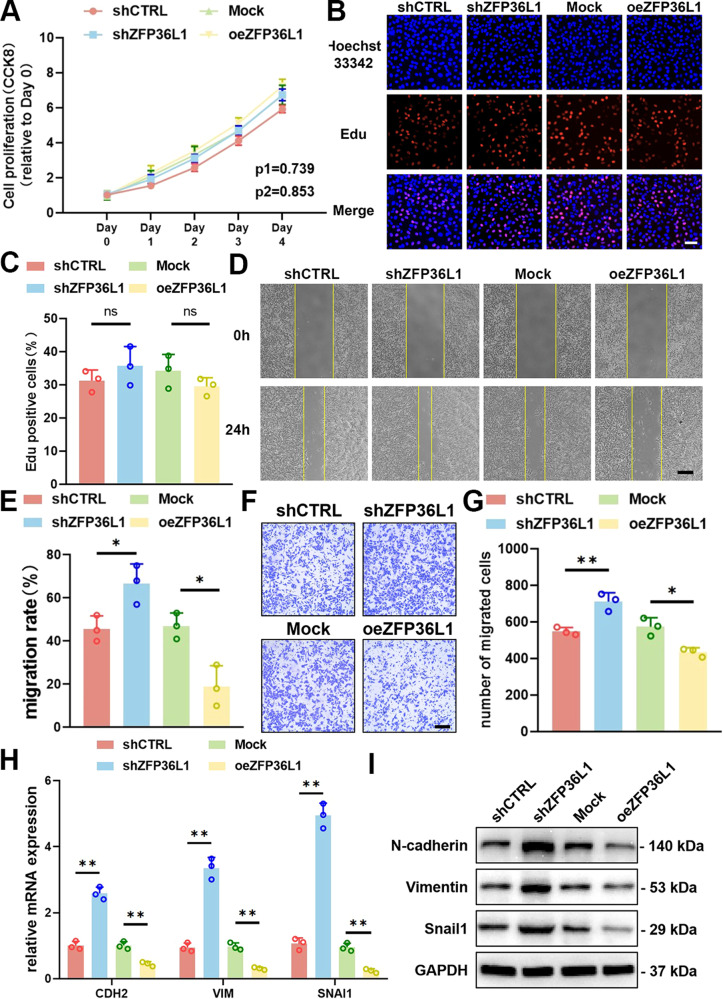
Effect of ZFP36L1 on the proliferation, migration and EMT of 143B cells. **A**–**C** CCK8 assays (**A**) and EdU staining assays (**B**) were conducted to determine the proliferation rate of oeZFP36L1 or shZFP36L1 143B cells. The percentage of EdU-positive cells (**C**) was determined by ImageJ. Scale bar, 80 μm. **D**-**G** The migration of 143B cells was measured using a wound healing test (**D**) and Transwell migration assay (**F**). Quantitative analyses (**E**, **G**) were carried out with ImageJ. Scale bar, 150 μm. q-PCR assays (**H**) and WB (**I**) were performed to determine the role of ZFP36L1 in regulating EMT-associated gene expression in 143B cells. The data are shown as means ± SEMs; *P < 0.05, **P < 0.01.

To explore the role of ZFP36L1 in OS cells, we performed CCK-8 assays, EdU staining assays, wound healing assays, and Transwell migration assays. The results showed that OS cells transfected with shZFP36L1 or oeZFP36L1 had a proliferative capacity that was comparable to that of control cells (Fig. 2A–C; S2A–C). However, both shZFP36L1 143B and U2OS cells exhibited greater migration than control cells, as shown by wound healing assays and Transwell migration assays. Accordingly, oeZFP36L1 inhibited OS cell migration (Fig. 2D–G; S2D–G). These results suggested that ZFP36L1 suppresses the migration of OS.

Epithelial-mesenchymal transition (EMT) is an important regulator of tumor migration and metastasis [14, 15]. We then examined whether ZFP36L1 regulated OS migration by regulating EMT. The expression of CDH1/E-cadherin was quite low in 143B and U2OS cells, as shown by q-PCR or WB (data not shown). However, markers of the mesenchymal state (CDH2/N-cadherin, VIM/Vimentin, SNAI1/Snail1) were detectable. The results showed that shZFP36L1 increased mesenchymal marker expression, while oeZFP36L1 decreased mesenchymal marker expression in OS cells (Fig. 2H, I; S2H, I). Taken together, these results suggest that ZFP36L1 decreases OS migration via the inhibition of EMT.

### ZFP36L1 inhibited TGF-β signaling in OS cells

**Fig. 3:**
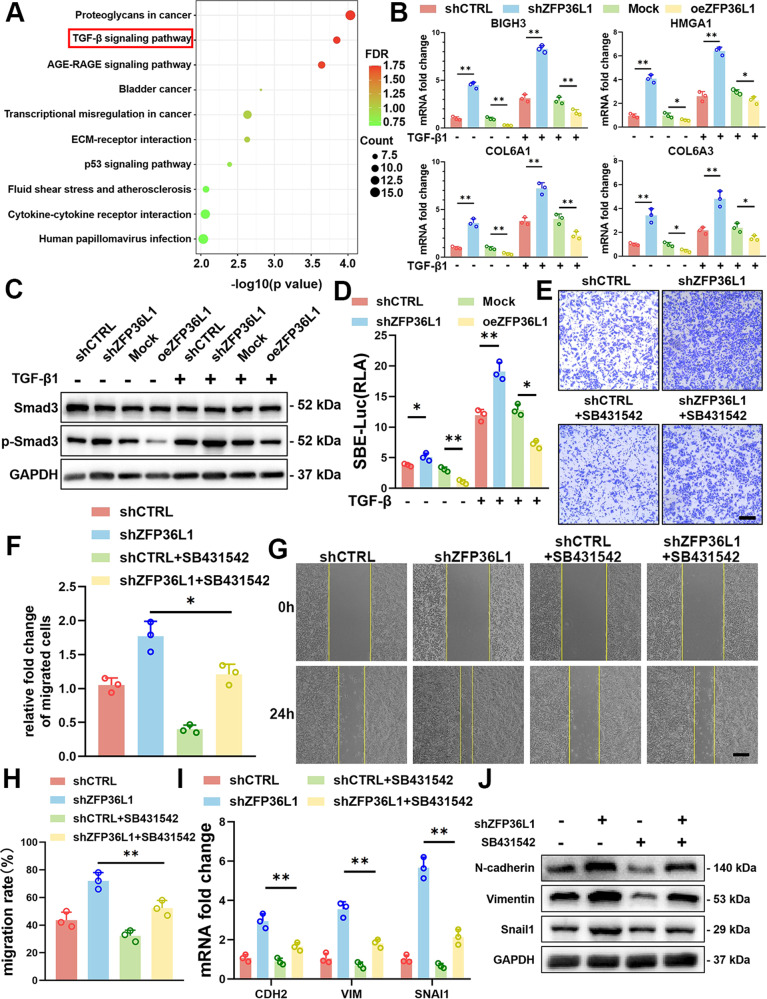
ZFP36L1 inhibited 143B cell migration by regulating the TGF-β signaling pathway. **A** KEGG pathway analysis of differentially expressed genes (log_2_(oeZFP36L1/mock) ≥ 1) identified by transcriptome sequencing of mock and oeZFP36L1 143B cells. **B** q-PCR analysis of gene expression downstream of the TGF-β signaling pathway in 143B cells stimulated with or without TGF-β1. **C** WB analysis of Smad3 phosphorylation levels in 143B cells. **D** SBE luciferase activity was measured to show the activation of the TGF-β signaling pathway. **E**–**H**, Transwell migration assays (**E**) and wound healing assays (**G**) were performed to determine the migration ability of 143B cells with or without SB431542 stimulation. Quantitative analyses (**F**, **H**) were carried out with ImageJ. Scale bar, 150 μm. q-PCR assays (**I**) and WB (**J**) were carried out to measure EMT-associated gene expression in 143B cells with or without SB431542 stimulation. The data are shown as means ± SEMs; *P < 0.05, **P < 0.01.

To delineate the functional implications of ZFP36L1 in OS, we performed transcriptome sequencing to investigate changes in gene expression in shZFP36L1 and oeZFP36L1 143B cells. According to Kyoto Encyclopedia of Genes and Genomes pathway analysis, gene set enrichment analysis and gene ontology biological process analysis, differentially expressed genes were significantly enriched in gene sets that are involved in the TGF-β signaling pathway, which crucially contributes to the modulation of cell migration capability (Fig. 3A and S3A-C). We also examined the expression of several genes that are downstream of the TGF-β signaling pathway (BIGH3, HMGA1, COL6A1, and COL6A3) in 143B cells with/without TGF-β1 stimulation. The results showed that BIGH3, HMGA1, COL6A1, and COL6A3 expression was increased by shZFP36L1 but decreased by oeZFP36L1 (Fig. 3B). These results indicate that TGF-β signaling might be activated in shZFP36L1 OS cells. The biological actions of TGF-β are mediated by high-affinity serine-threonine kinase receptors (TβR-I and TβR-II) and subsequent activation of the SMAD-dependent signaling cascade, which leads to the transcriptional regulation of a set of gene promoters containing the SMAD-binding element (SBE), which is also called the CAGA box [16, 17]. To examine whether TGF-β signaling is regulated by ZFP36L1, we analyzed p-SMAD3 levels and SBE luciferase activity (known to reveal TGF-β signaling) in shZFP36L1 and oeZFP36L1 OS cells. The results showed that the protein level of p-SMAD3 and SBE-luciferase activity were increased in shZFP36L1 OS cells but were decreased in oeZFP36L1 OS cells (Fig. 3C, D and S4A, B). These results indicate that ZFP36L1 inhibited the TGF-β signaling pathway in OS cells.

To further examine whether ZFP36L1 regulates OS cell migration through TGF-β signaling, we performed wound healing and Transwell migration assays. The results showed that the shZFP36L1-induced increase in OS cell migration was significantly reversed by SB431542 (a TGF-β receptor kinase inhibitor) treatment (Fig. 3E–H and S4C-F). Accordingly, the shZFP36L1-induced increase in mesenchymal marker expression was also attenuated by SB431542 treatment (Fig. 3I, J and S4G-I). These results suggest that shZFP36L1 increased OS cell migration via TGF-β signaling activation.

### ZFP36L1 regulated the SDC4-TGF-β signaling loop

**Fig. 4:**
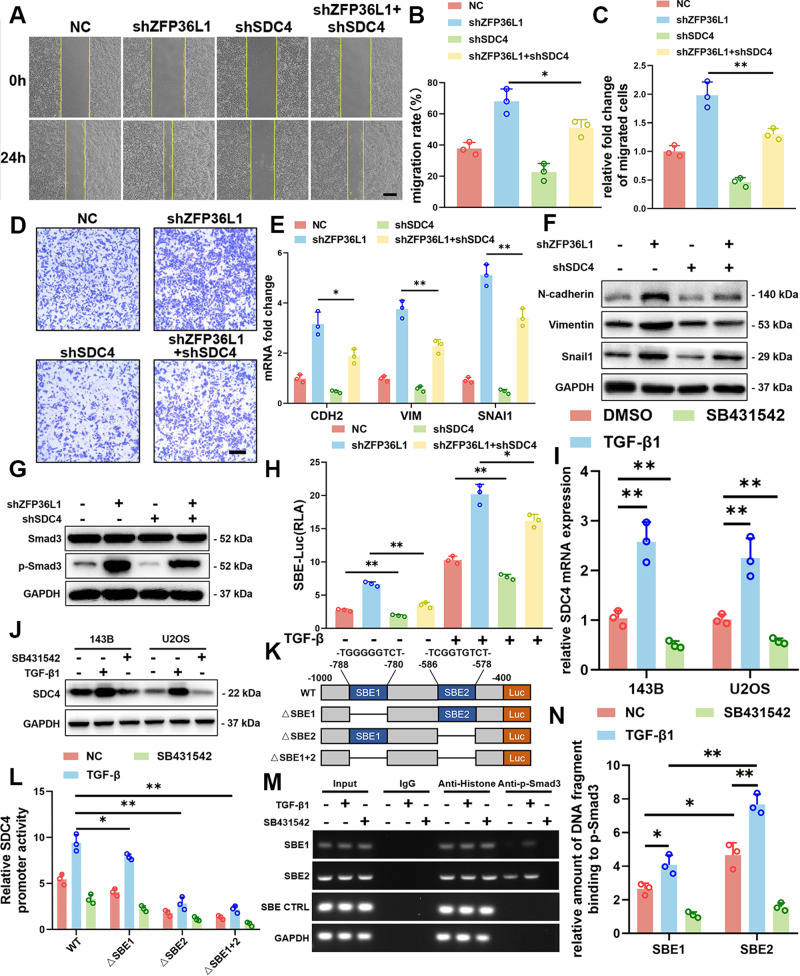
ZFP36L1 inhibited the migration ability of 143B cells through the SDC4-TGF-β signaling loop. **A**–**D**, Wound healing assays (**A**) and Transwell migration assays (**D**) were performed to show the relationship between ZFP36L1 and SDC4 in regulating the migration of 143B cells. Quantitative differences (**B**, **C**) were analyzed with ImageJ. Scale bar, 150 μm. q-PCR assays (**E**) and WB (**F**) were carried out to show the relationship between ZFP36L1 and SDC4 in regulating EMT-associated gene expression in 143B cells. **G**, **H** WB and SBE-luciferase activity assays were performed to indicate the relationship between ZFP36L1 and SDC4 in regulating the TGF-β signaling pathway in 143B cells. The role of the TGF-β signaling pathway in regulating SDC4 expression in 143B cells was examined using q-PCR assays (**I**) and WB (**J**). **K** Schematic representation of the predicted SBE in the SDC4 gene promoter and construction of the corresponding mutant promoters. **L** Relative luciferase activity was assayed to show SDC4 promoter activity after TGF-β1 or SB431542 stimulation. **M** Chips of predicted SBEs within the SDC4 promoter region were examined by PCR. The promoter without SBE (SBE CTRL) and the promoter region of GAPDH were used as negative controls; histone antibody or rabbit IgG was used as an assay control. **N** ChIP results were quantitatively analyzed by qRT‒PCR. The data are shown as means ± SEMs; *P < 0.05, **P < 0.01.

To explore the downstream regulatory of ZFP36L1, we examined the expression of genes associated with the TGF-beta signaling pathway based on our RNA-seq in 143B cells. However, shZFP36L1 cells did not exhibit the expected upregulation of these genes (Fig. S5A). This discrepancy prompted us to explore alternative regulators of the TGF-β signaling pathway. We systematically examined the expression patterns of the top 10 genes that displayed the most significant changes in our RNA-seq data, leading us to identify Syndecan-4 (SDC4) (Fig. S5B–D). SDC4 is a cell surface proteoglycan that regulates cancer progression and has a positive correlation with TGF-β signaling pathway [18–20]. However, its role in OS migration remains unknown. We established shSDC4 and oeSDC4 OS cell lines (Fig. S6) and showed that SDC4 promoted cell migration (Fig. S7A–D) and EMT (Fig. S7E, F) in OS cells. We also found that the shZFP36L1-induced increases in cell migration and EMT were attenuated by shSDC4 (Fig. 4A–F and S7G, H). These results suggested that ZFP36L1 regulated OS cell migration by inhibiting SDC4 expression.

Both SDC4 expression and TGF-β activation are involved in ZFP36L1-mediated cell migration; thus, we explored their relationship. As shown in Fig. 4G, H, shSDC4 significantly inhibited TGF-β signaling in OS cells with and without shZFP36L1. Interestingly, inhibition of TGF-β signaling also reduced SDC4 expression (Fig. 4I, J). These results suggested a regulatory loop between SDC4 and TGF-β signaling.

### TGF-β signaling promoted SDC4 expression through p-SMAD3

SMAD3 is a transcriptional modulator that is activated by TGF-β and highly expressed in OS. According to ReMap 2022 [21], SMAD3 bound to the SDC4 promoter region. We used JASPAR 2022 [22] to search the SDC4 promoter, i.e., the − 1000 bp to 0 bp sequence upstream of the transcription start site, and found candidate SMAD3 binding sequences (SBE1 and SBE2). To analyze the SDC4 promoter motifs that are required for TGF-β-mediated expression, we assessed SDC4 promoter luciferase constructs with deletions in the SBE1 and/or SBE2 motifs (Fig. 4K). TGF-β-induced SDC4 promoter activation was substantially reduced by deletion of the SBE2 motif, although SBE1 deletion decreased SDC4 promoter activity to a lesser extent (Fig. 4L). Direct binding of SMAD3 to the SDC4 promoter was also monitored by anti-SMAD3 ChIP, followed by PCR for the SBE motifs in the SDC4 promoter. ChIP revealed enriched SMAD3 binding within regions of the putative SBE motifs that were predicted by JASPAR. Moreover, the binding of SMAD3 to SBE1 and SBE2 was increased by TGF-β1 treatment, with SBE2 showing more enrichment than SBE1 (Fig. 4M, N). These results indicate that TGF-β1 mediates SDC4 transcription.

### SDC4 regulated TGF-β signaling by protecting against TGFBR3 cleavage

**Fig. 5:**
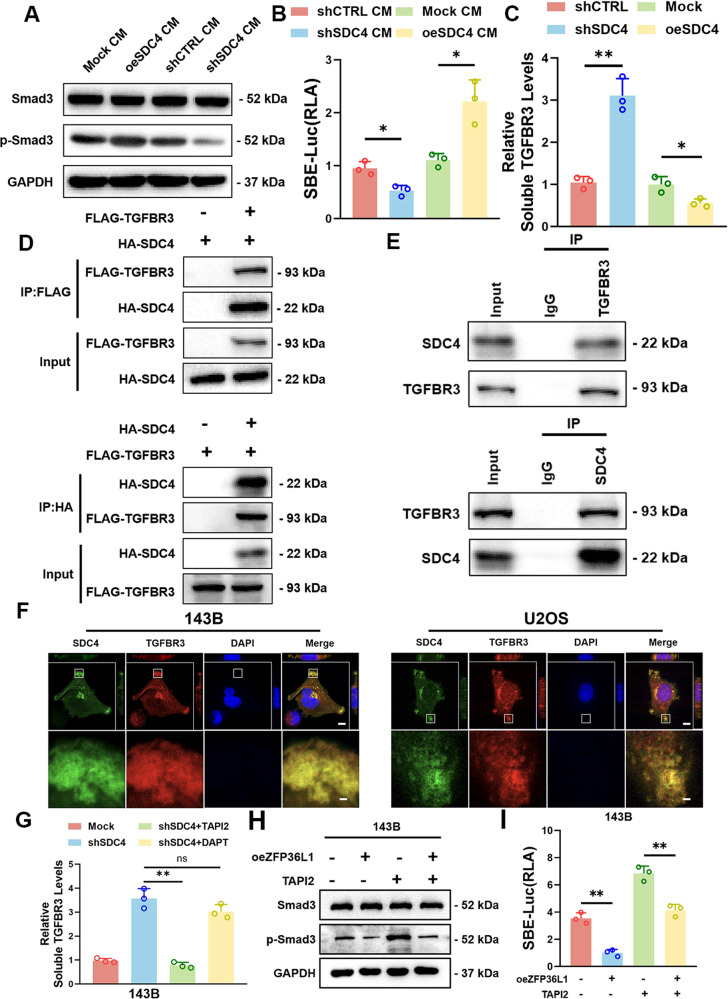
Interaction between SDC4 and TGFBR3 protected against TGFBR3 cleavage by MMP. WB (**A**) and SBE-luciferase activity assays (**B**) were conducted to determine the role of SDC4 in regulating the TGF-β signaling pathway via the extracellular pathway in 143B cells. **C** ELISA tests were performed to measure the level of sTGFBR3 in the CM of oeSDC4 or shSDC4 143B cells. IP assays were carried out to reveal the interaction between SDC4 and TGFBR3 in exogenous (**D**) and endogenous (**E**) ways in 143B cells. **F** Immunofluorescence assay showed the colocalization of SDC4 and TGFBR3 on the cell surface in 143B cells or U2OS cells. Upper scale bar, 10 μm. Lower scale bar, 1 µm. **G** ELISA was conducted to measure TGFBR3 cleavage regulated by TAPI2 or DAPT in 143B cells. WB (**H**) and SBE-luciferase activity (**I**) assays were conducted to elucidate the role of TAPI2 in the modulation of the TGF-β signaling pathway attenuated by oeZFP36L in 143B cells. The data are shown as means ± SEMs; *P < 0.05, **P < 0.01.

shSDC4 inhibited TGF-β signaling (Fig. 4G, H and S8). However, the function of SDC4 in TGF-β signaling remains unknown. We first treated control cells with conditioned media (CM) from shSDC4 and oeSDC4 cells. The results showed that TGF-β signaling was significantly activated by oeSDC4 CM but inhibited by shSDC4 CM (Fig. 5A, B), indicating that SDC4 promotes TGF-β signaling through extracellular factors. We then analyzed the contents of TGF-β family members in the CM. Unexpectedly, the TGF-β1, BMP2, BMP4 and BMP6 levels were not altered by shSDC4 or oeSDC4 (Fig. S9A).

TGFBR3 can undergo ectodomain shedding, releasing a soluble form of TGFBR3 (sTGFBR3). sTGFBR3 inhibits TGF-β signaling via extracellular sequestration of TGF-β ligands [9]. ELISA showed that the sTGFBR3 contents in CM were decreased by oeSDC4 but increased by shSDC4 (Fig. 5C). However, qRT‒PCR showed that total TGFBR3 expression was not changed by shSDC4 or oeSDC4 (Fig. S9B). These results indicated that SDC4 inhibits TGFBR3 cleavage.

Intriguingly, we noticed that SDC4 interacted with TGFBR3. As shown in Fig. 5D, E, a direct interaction between SDC4 and TGFBR3 was observed by immunoprecipitation assay. Furthermore, confocal microscopy revealed that TGFBR3 and SDC4 colocalized on the cell surface (Fig. 5F). Furthermore, shSDC4-induced TGFBR3 cleavage was significantly inhibited by TAPI-2, which is a broad-spectrum inhibitor of matrix metalloprotease (MMP), but not by the γ-secretase inhibitor DAPT (Fig. 5G and S10A). We also found that TAPI-2 effectively attenuated inhibition of TGF-β signaling pathway induced by oeZFP36L1 or shSDC4 in OS cells (Fig. 5H, I and S10B–E). Together, these data demonstrate that SDC4 interacts with TGFBR3 and inhibits TGFBR3 cleavage by MMP.

### ZFP36L1 promoted SDC4 mRNA degradation through the ARE element

**Fig. 6:**
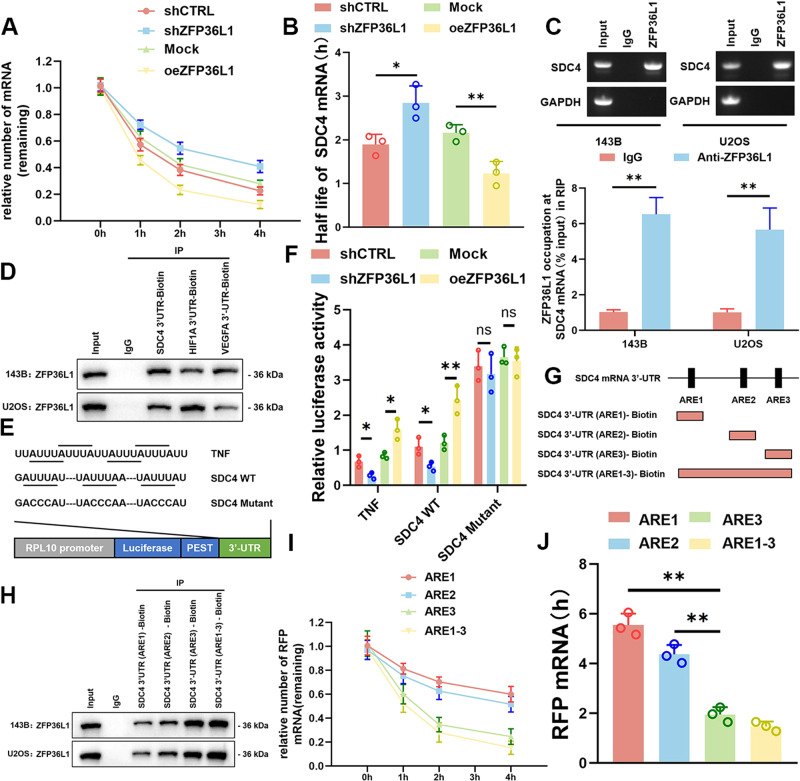
ZFP36L1 accelerated SDC4 mRNA degradation by binding to the ARE element. mRNA stability assays (**A**) were performed to determine the stability of SDC4 mRNA that was regulated by ZFP36L1, and the half-life of SDC4 mRNA (**B**) was analyzed. RIP assays (**C**) and RNA pull-down assays (**D**) were carried out to confirm the binding relationship between ZFP36L1 and SDC4 mRNA. **E** Schematic representation of the predicted AREs in the 3’UTR of SDC4 mRNA (NM_002999.4) and construction of corresponding reporter plasmids. The 3’UTR in TNF mRNA (NM_000594.4) was used as a positive control. **F** Renilla luciferase activity assays were conducted to determine the role of AREs in the 3’UTR of SDC4 mRNA in the regulation of mRNA decay. RIP assays (**G**) and RNA pull-down assays (**H**) confirmed that ARE3 was the most important element for ZFP36L1 binding. **I**, **J**, RFP plasmids were constructed by fusing 3 AREs with RFP genes. mRNA stability assays (**I**) were performed to determine the function of AREs in regulating RFP mRNA decay, and the half-life of RFP mRNA (**J**) was calculated. The data are shown as means ± SEMs; *P < 0.05, **P < 0.01.

Since ZFP36L1 is a negative regulator of mRNA stability and SDC4 mRNA was reported to be a potential target of ZFP36L1 [8], the half-life of SDC4 mRNA was measured in 143B cells. As shown by the mRNA stability assay, oeZFP36L1 increased the rate of SDC4 mRNA decay, while shZFP36L1 decreased the rate of SDC4 mRNA decay (Fig. 6A, B). Moreover, ZFP36L1 directly bound to SDC4 mRNA (Fig. 6C). ZFP36L1 recognizes ARE sequences in the 3’UTRs of RNA through its tandem zinc-fingers [5]. To determine whether ZFP36L1 binds to SDC4 mRNA through its 3’UTR, we constructed SDC4 3’UTR-Biotin and performed an RNA pull-down assay. The results showed that ZFP36L1 was pulled down by SDC4 3’UTR-Biotin from both 143B and U2OS cells (Fig. 6D). Taken together, these results suggest that ZFP36L1 interacts with the 3’UTR of SDC4 mRNA.

To demonstrate that ZFP36L1 mediates SDC4 mRNA degradation via ARE motifs in its 3’UTR, dual-luciferase assays were performed using a construct in which the Renilla-PEST reporter gene was fused to the entire 3’UTR sequence (WT or mutant) of SDC4 (Fig. 6E). The reporter plasmids were cotransfected with oeZFP36L1, shZFP36L1 or control plasmid into HEK293T cells. Overexpression of ZFP36L1 profoundly reduced Renilla luciferase activity while shZFP36L1 increased Renilla luciferase activity in the WT SDC4 group (Fig. 6F). However, oeZFP36L1 or shZFP36L1 did not alter Renilla luciferase activity in the mutant SDC4 group. These results indicated that ZFP36L1 regulates SDC4 mRNA decay through its ARE in the 3’UTR.

We also explored the function of the 3 AREs (ARE1, ARE2, and ARE3) in the 3’UTR of SDC4 mRNA. The results showed that ARE3 was the major ARE to which ZFP36L1 bound (Fig. 6G–J).

### Targeting the ZFP36L1-SDC4-TGF-β loop inhibited osteosarcoma lung metastasis

**Fig. 7:**
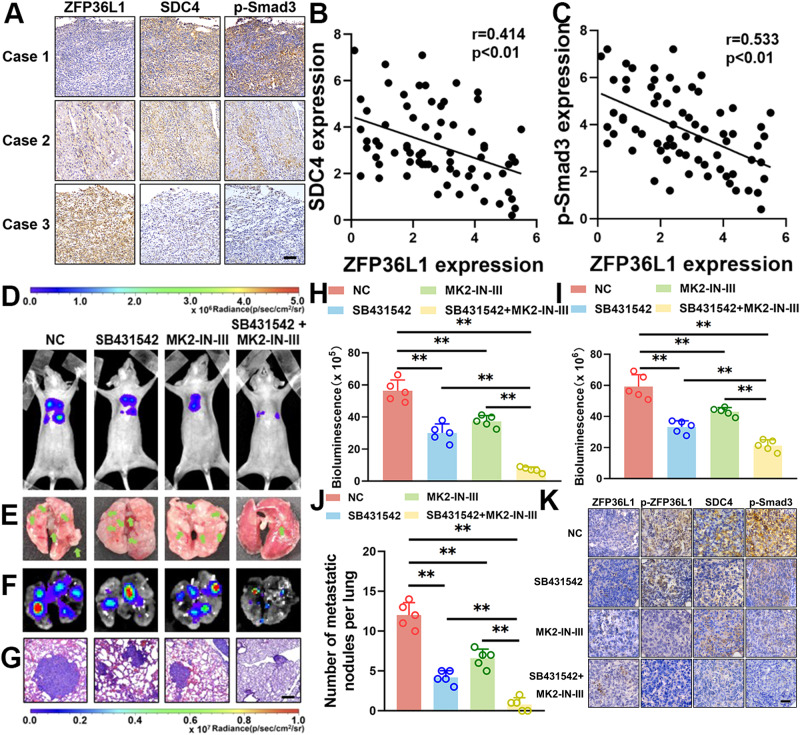
Impact of the ZFP36L1-SDC4-TGF-β loop on osteosarcoma lung metastasis in vivo. **A** Representative IHC images of ZFP36L1, SDC4 and p-SMAD3 in individual samples from among 70 OS specimens. Scale bar, 100 μm. Percentage of samples with high or low expression of ZFP36L1 compared to SDC4 (**B**) and p-SMAD3 (**C**). Lung metastasis of osteosarcoma was measured by in vivo bioluminescence (**D**), lung tumor counts (**E**), ex vivo bioluminescence (**F**) and HE staining (**G**). Scale bar, 120 μm. The bioluminescence levels of in vivo bioluminescence images **(H)** and ex vivo bioluminescence images **(I)** were analyzed by LivingImage software. **J** The number of lung tumors in different groups of mice was counted. **K** Representative osteosarcoma metastatic lesion in the lungs from different groups of mice were analyzed by IHC staining for ZFP36L1, p-ZFP36L1, SDC4, and p-SMAD3. Scale bar, 60 μm. The data are shown as means ± SEMs; *P < 0.05, **P < 0.01.

To determine whether the ZFP36L1, SDC4 and p-SMAD3 levels are correlated in OS, we collected 70 patient tumor tissue sections. Representative staining images with high or low levels of ZFP36L1, SDC4 and p-SMAD3 expression are shown in Fig. 7A. The scores of these images were evaluated by systematically analyzing the IHC staining results. As expected, statistically significant negative correlations between ZFP36L1 and SDC4 (Fig. 7B) and ZFP36L1 and p-SMAD3 (Fig. 7C) were observed. These findings suggest that the ZFP36L1-SDC4-TGF-β axis is associated with the clinicopathological features of OS.

We reasoned that targeting the ZFP36L1-SDC4-TGF-β axis may inhibit OS lung metastasis. As shown in Fig. 7D–K, SB431542 significantly inhibited OS lung metastasis. A previous study showed that MAPKAPK2 phosphorylates ZFP36L1, inhibiting its ability to degrade mRNA [23]. We treated mice with MK2 inhibitor III, which is an inhibitor of MAPKAPK2. The results showed that the level of p-ZFP36L1 was decreased, which was accompanied by lower SDC4 expression and reduced OS lung metastasis in vitro and in vivo (Fig. 7D–K, S11, S12); these results suggested that MK2 inhibitor III increased the ability of ZFP36L1 to degrade SDC4 mRNA. Furthermore, we treated mice with both SB431542 and MK2 inhibitor III. As expected, the combination treatment inhibited OS lung metastasis dramatically more than SB431542 or MK2 inhibitor III treatment alone. These results suggested that the combination of a MAPKAPK2 inhibitor and a TGF-β signaling inhibitor might be a promising therapeutic approach to treat OS lung metastasis.

## Discussion

**Fig. 8:**
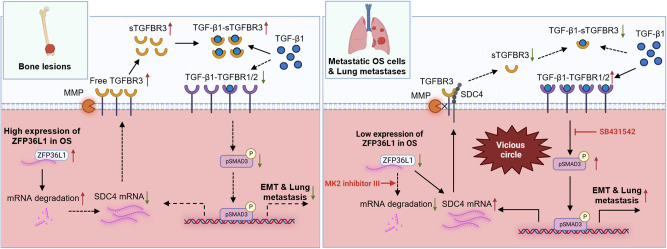
Blocking the ZFP36L1-SDC4-TGF-β loop inhibited osteosarcoma EMT and lung metastasis. In bone lesions, the high expression of ZFP36L1 expedited SDC4 mRNA degradation and reduced SDC4 interacted with TGFBR3, leading to increased free TGFBR3 cleavage by MMP. The increased soluble TGFBR3 (sTGFBR3) subsequently inhibited activation of TGF-β signaling pathway via blocking TGF-β1 binding to TGFBR1/2. Conversely, in metastatic OS cells and lung metastases, the low expression of ZFP36L1 reduced SDC4 mRNA degradation, leading to an abnormal accumulation of SDC4. The interaction between SDC4 and TGFBR3 protected against TGFBR3 cleavage by MMP, resulting in a decrease in extracellular sTGFBR3. The decrease in sTGFBR3 facilitated the activation of the TGF-β signaling pathway by enhancing the binding of TGF-β1 to TGFBR1/2, ultimately resulting in osteosarcoma EMT and lung metastasis. Intriguingly, activation of the TGF-β signaling pathway further upregulated SDC4 expression, which caused a vicious circle. Targeting the ZFP36L1-SDC4-TGF-β loop with MK2 inhibitor III or SB431542 effectively inhibited osteosarcoma EMT and lung metastasis.

Recently, many RBPs have been shown to regulate key cancer-related processes, such as cell proliferation, apoptosis, and metastasis [13]. Moreover, targeting RBPs (such as HuR [24–26], LIN28 [27, 28], MSI [29] and ALKBH5 [30, 31]) has been identified as an effective therapeutic approach for cancer treatment. In this study, we reveal the cancer-inhibitory effect of the RBP ZFP36L1 in OS. Low expression of ZFP36L1 promotes the SDC4-TGF-β signaling loop to increase OS EMT and lung metastasis. An increase in ZFP36L1 expression or function inhibits the feedback loop between SDC4 expression and TGF-β signaling, representing a promising treatment for OS lung metastasis (Fig. 8).

The function of ZFP36L1 in carcinoma has been well studied. In many carcinomas (e.g., liver carcinoma [32], lung carcinoma [33] and colorectal carcinoma [34]), ZFP36L1 represses oncogenic protein expression and tumor progression, whereas in gastric carcinoma [35], ZFP36L1 promotes tumor progression. These results suggest that ZFP36L1 has different functions in different types of carcinomas. However, the function of ZFP36L1 in sarcoma remains unknown. In this study, we showed that OS cells also expressed ZFP36L1. By knockdown and overexpression of ZFP36L1, we found that ZFP36L1 suppressed EMT and metastasis of OS cells, indicating a tumor-suppressing role of ZFP36L1 in sarcoma.

ZFP36L1 acts as an adapter protein and interacts with RNA degradation complexes to participate in ARE-mediated RNA decay. To identify direct downstream targets of ZFP36L1, Xin-Yi Loh and colleagues performed an RNA pull-down screen of ZFP36L1 in bladder cancer [8]. The results showed that ZFP36L1 interacted with the 3’UTRs of numerous oncogenic transcripts that are involved in the cell cycle and hypoxic signaling pathways, leading to the degradation of these mRNAs and cell cycle arrest. However, experiments such as RNA pull-down screens of ZFP36L1 in OS are lacking. By knocking down ZFP36L1 in OS cells, we found that ZFP36L1 expression had little effect on the cell cycle. Instead, ZFP36L1 regulated the EMT and migration of OS cells. These results suggested that ZFP36L1 had different target genes in OS cells compared with bladder or other cancer cells.

SDC4 is a transmembrane proteoglycan that is involved in numerous signaling processes, such as sequestration of growth factors, regulation of Rac1 activity, and regulation of FAK activity [36]. Previous studies have suggested that SDC4 regulates TGF-β signaling [37], but the mechanism is unclear. Here, we showed that SDC4 directly participated in TGF-β signaling through the inhibition of TGFBR3 cleavage, suggesting that SDC4 is an important regulator of TGF-β signaling.

SDC4 is ubiquitously expressed in tumors. Ki Yong Na and colleagues showed that strong SDC4 expression was associated with the occurrence of distant metastasis and large tumor size in OS, indicating that the increased expression of SDC4 accounts for more aggressive clinical behavior in OS [38]. We also showed that SDC4 overexpression increased OS cell migration. However, the mechanism by which OS cells maintain high levels of SDC4 expression is unclear. In this study, we demonstrated that TGF-β signaling activation in OS increased SDC4 expression through SMAD3. Moreover, low expression of ZFP36L1 in OS decreased the degradation of SDC4 mRNA. Therefore, these two mechanisms maintain high SDC4 expression in OS.

TGFBR3 was originally thought to function as a TGF-β coreceptor that serves only to sequester and present ligands to TGFBR2 [10]. It was later shown that TGFBR3 can undergo ectodomain cleavage, releasing a soluble form of TGFBR3 (sTGFBR3), which sequesters TGF-β ligands extracellularly and therefore inhibits TGF-β signaling [11]. The specific mechanism underlying this cleavage is still not well characterized. In this study, we found that SDC4 inhibited the cleavage of TGFBR3, suggesting that SDC4 plays an important role in the process of TGFBR3 cleavage. However, the specific mechanism by which SDC4 protects TGFBR3 from cleavage remains to be further explored.

TGF-β signaling is an important target for the treatment of OS [39]. Many studies have shown that TGF-β signaling is abnormally activated in OS and closely related to processes that are involved in OS progression, such as proliferation and metastasis [40–42]. Lamora, A. et al. showed that the levels of TGF-βs are increased in the sera of patients with OS compared to the sera of healthy donors [43]. One important reason is that latent precursor molecules of TGF-β1 that are deposited in the bone matrix are activated and released by OS-educated osteoclasts [44]. Treatment with bisphosphonates to inhibit osteoclast activity is thought to decrease TGF-β1 levels and attenuate the vicious cycle between OS cells and bone. However, clinical trials have yet to demonstrate the benefits of bisphosphonate treatment in patients with OS [45], indicating the complexity of TGF-β signaling regulation in OS. In our study, we found that OS has a mechanism that allows the efficient utilization of TGFβ. High SDC4 expression protects TGFBR3 from cleavage and thus increases the effective concentration of TGF-β1 and activates the TGF-β signaling pathway.

During the last decade, numerous strategies for targeting TGF-β signaling have been used in preclinical or clinical applications [39]. However, these approaches have not shown spectacular success in clinical trials. Further study and treatment strategies that target the TGF-β signaling pathway are needed. In our study, the combination of a MAPKAPK2 inhibitor and a TGF-β receptor kinase inhibitor repressed TGF-β signaling and OS lung metastasis to a better extent than either single treatment alone. These results suggested that targeting the crosstalk between TGF-β signaling and other signaling pathways might represent a new strategy for OS treatment.

## Materials and methods

### Tissue collection

All paraffin-embedded OS tissues that were used in this study were obtained from patients with OS at the Eighth Affiliated Hospital, Sun Yat-sen University (Shenzhen, China). The study was approved by the Ethics Committee of The Eighth Affiliated Hospital, Sun Yat-sen University. All patients were provided informed consent and clinical information is presented in Table S1.

### Immunohistochemistry (IHC) staining

Formalin-fixed paraffin-embedded samples were obtained from individual patients with OS. The sections were incubated with primary antibodies against ZFP36L1 (Solarbio, #K110803P), Vimentin (CST, #5741), N-cadherin (CST, #13116), p-ZFP36L1 (SAB, #11705), SDC4 (CST, #12236) and p-Smad3 (CST, #9520). Immunostaining was performed using the SP Rabbit & Mouse HRP Kit (ComWin Biotech, China) according to the manufacturer’s instructions. Images were captured by microscopy.

The staining intensity was evaluated independently by two pathologists, and the scores were quantified according to the percentage of positive cells and staining intensity. The percentage scores were determined as follows: 0, no positive cells; 1, ≤10% positive cells; 2, 10–50% positive cells; and 3, >50% positive cells. The staining intensity scores were determined as follows: 0, no staining; 1, weak staining; 2, moderate staining; and 3, dark staining. The comprehensive score = the percentage score x the staining intensity score. An ZFP36L1 expression score ≥3 indicated a high level, whereas a score <2 indicated a low level; N-cadherin and Vimentin expression scores ≥4 indicated a high level, whereas scores <2 indicated a low expression [46].

### Cell lines and culture conditions

All OS cell lines (143B, U2OS, and Saos-2 cell lines) and 293 T cells were purchased from American Type Culture Collection (ATCC, Manassas, VA, USA) and cultured according to the instructions from ATCC. All cell lines were regularly tested for Mycoplasma to ensure they were authenticity.

### Generation of stable knockdown and overexpression cell lines

Plasmids (pLKO.1-shZFP36L1, pLKO.1-shSDC4, pLVX-ZFP36L1-FLAG, pLVX-SDC4-HA and pLVX-TGFBR3-FLAG) were constructed by Fubio Biotechnology (Suzhou, China). The generation of knockdown and overexpression cell lines was manufactured as previously described [46]. The target sequences were as follows: shZFP36L1#1: 5’- GCT CGC GAG ACA GCC GCT TCC -3’; shZFP36L1#2: 5’- GCT TCC GAG ACC GCT CCT TCT -3’; shSDC4#1: 5’- GCC CGG GCA GGA ATC TGA TGA -3’; and shSDC4#2: 5’- GCA GGG CAG CAA CAT CTT TGA -3’.

### Animal study

Six- to eight-week-old male BALB/c nude mice were used for the animal studies, which were approved by the Sun Yat-sen University Laboratory Animal Care and Use Committee (Guangzhou, China). To establish the metastatic OS xenograft model, mice were randomly divided into groups, each consisting of five mice and 2 × 10^6^ 143B cells stably expressing luciferase were intravenously injected into the tail vein of the mice. Four weeks later, luciferase activity was measured by scanning the mice with a Xenogen IVIS 200 imaging system. The mice were euthanized 32–40 days after cell implantation, and the lungs were harvested for ex vivo bioluminescence, metastatic lesion quantification, and H&E staining to determine tumor burden.

The TGF-β inhibitor SB431542 (Beyotime, China) and MK-2 inhibitor III (Merck, Germany) were delivered intraperitoneally every day for 4 weeks at concentrations of 10 mg/kg and 20 mg/kg respectively.

### RNA extraction and quantitative real-time PCR (qRT-PCR)

Total RNA was extracted with an RNA-Quick Purification Kit (ES Science, China) according to the manufacturer’s instructions. RNA (1–2 mg) was reverse-transcribed into cDNA using Evo M-MLV RT Premix for q-PCR kit (Accurate Biology, China). Quantitative reverse transcription PCR was performed using the SYBR Green Pro Taq HS q-PCR Kit (Accurate Biology, China). All the primers that were used for qRT‒PCR in this research are listed in Table S2.

### RNA sequencing (RNA-seq)

RNA sequencing assays were carried out by Beijing Genomics. Briefly, the RNA concentration and purity of each sample were quantified using the Standard Sensitivity RNA Analysis Kit (15 nt) (Agilent, USA). The RNA samples were sequenced based on DNBSEQ Transcriptome. The raw data were filtered with SOAPnuke (v1.5.2) to obtain clean data, which were mapped to the reference genome (GCF 000001405.39 GRCh38.p13 of Homo sapiens) by HISAT (v2.1.0) and mapped to the assembled unique gene by Bowtie2 (v12.2.5). The expression levels of genes were calculated via RSEM (v1.2.8).

### Western blotting (WB)

Whole-cell lysates were extracted with RIPA lysis buffer (Beyotime, China). Equal amounts of protein samples were separated by 8–12% SDS‒PAGE and transferred to polyvinylidene difluoride membranes (0.45 mm, Merck Millipore, USA). After blocking in 5% skim milk (Wako, Japan), the membranes were incubated at 4 °C overnight with primary antibodies against ZFP36L1 (Solarbio, #K110803P), p-ZFP36L1 (SAB, #11705), Vimentin (CST, #5741), N-cadherin (CST, #13116), Snail1 (CST, #3879), Smad3 (CST, #9523), p-Smad3 (CST, #9520), SDC4 (CST, #12236) and GAPDH (CST, #5174). The membranes were washed 3 times with TBST buffer followed by incubation with horseradish peroxidase (HRP)-conjugated antibody (Boster, China). HRP activity was detected using an ECL detection system (Bio-Rad, USA), and the images were quantified using ImageJ software.

### Cell proliferation assay

Cell proliferation was measured by Cell Counting Kit-8 (CCK-8) assay and 5-ethynyl-2’deoxyuridine (EdU) assay. For the CCK8 assay, 1 × 10^3^ 143B cells and 2 × 10^3^ U2OS cells were plated in 96-well plates. 10 µl of CCK-8 solution (Beyotime, China) was added to each well at the indicated times (0, 24, 48, or 72 h) followed by incubation at 37 °C for 2 h. Cell viability was monitored by measuring the absorbance at 450 nm with a Varioskan LUX microplate reader (Thermo Fisher, USA). The EdU assay was performed using the BeyoClick™ EdU Cell Proliferation Kit with Alexa Fluor 555 (Beyotime, China) according to the manufacturer’s instructions.

### Wound-healing assay

A total of 5 × 10^4^ 143B or 1 × 10^5^ U2OS cells were seeded in 12-well plates overnight. When the cells reached 90% confluence, the medium was replaced with FBS-free DMEM. Scratch wounds were created in the cell monolayers with sterile 200 μL pipette tips. The area of each scratch was photographed at the indicated time and measured by ImageJ.

### Transwell migration assays

OS cell migration was assessed using 24-well plate-sized Transwell compartments with a pore size of 8 μm (Corning Falcon, USA). A total of 1 × 10^4^ 143B cells or 2 × 10^4^ U2OS cells suspended in 200 µl DMEM were seeded in the upper compartments, and 600 μl DMEM with 10% FBS was added to the lower compartments. After culturing for 24 h at 37 °C, the remaining cells in the upper compartments were removed, and the cells that migrated to the filter were fixed with 4% paraformaldehyde, stained with a 0.1% crystal violet solution, photographed under a microscope and counted by ImageJ.

### Immunoprecipitation (IP)

143B cells were lysed with 500 μL of IP lysis buffer and incubated with BeyoMag™ Protein A + G magnetic beads (Beyotime, China) and primary antibodies against SDC4 (CST, #12236), TGFBR3 (CST, #5544), HA-tag (CST, #2367) or FLAG-tag (CST, #14793) at 4 °C. After overnight incubation, the beads were washed three times and boiled with SDS loading buffer for further immunoblotting.

### Immunofluorescence (IF)

143B cells and U2OS cells were plated in confocal dishes at an appropriate density. Cells were fixed with 4% paraformaldehyde, treated with 0.5% Triton X-100, blocked with 1x goat serum and incubated with the indicated primary antibody overnight. Then, the cells were incubated with secondary antibodies for 1 h and counterstained with DAPI for another 10 min. All images were captured using an LSM 5 Exciter confocal imaging system (Carl Zeiss, Germany).

### Chromatin immunoprecipitation (ChIP) assay

ChIP assays were conducted using a SimpleChIP enzymatic ChIP kit (CST, USA) according to the manufacturer’s instructions. Chromatin supernatants were immunoprecipitated with antibodies against p-Smad3 (Abcam, #ab227223) and rabbit IgG. Then, DNA‒protein complexes were pulled down with protein A/G agarose. The precipitated DNA was purified and subjected to PCR amplification using specific primers. The primer sequences are listed in Table S3.

### Luciferase activity assay

The SDC4 promoter sequence and corresponding mutant sequence were cloned into a pGL3-basic luciferase reporter plasmid. A total of 1 ×10^4^ OS cells (143B or U2OS cells) were seeded in 48-well plates and cotransfected with the SBE-luciferase reporter plasmid and Renilla luciferase plasmid using Lipofectamine 3000 (Thermo, USA). After 48 h, luciferase activity was measured using a Dual Luciferase Reporter Assay kit (Promega, USA) according to the manufacturer’s instructions.

### Enzyme-linked immunosorbent assay (ELISA)

The 143B and U2OS OS cell lines were plated in a 6-well plate at densities of 2 × 10^5^ and 4 × 10^5^, respectively. After 24 h, the medium was replaced with 2 ml fresh DMEM supplemented with 10% fetal bovine serum, and the cells were incubated for another 24 h. The levels of soluble TGF-β1, BMP2, BMP4, BMP6 and TGFBR3 in the supernatants were quantified using a Human TGF-β1/BMP2/BMP4/BMP6 ELISA Kit (ABclonal, USA) and Human TGFBR3 ELISA Kit (Biotechwell, China) respectively according to the manufacturer’s instructions. The signals were detected using a Varioskan LUX microplate reader (Thermo Fisher, USA) at 498 nm.

### mRNA decay assay

Equal numbers of 143B cells were plated in 12-well plates and incubated overnight. Actinomycin D (Abmole, China) was added to each well at a concentration of 30 µg/ml, and total RNA was extracted using an RNA-Quick Purification Kit (ES Science, China) at the indicated times. Equal amounts of RNA were reverse-transcribed into cDNA, and gene expression was quantified by RT‒qPCR, with GAPDH as the internal control.

### RNA-binding protein immunoprecipitation (RIP) assay

The RIP assay for ZFP36L1 was performed with an RNA Immunoprecipitation Kit (Geneseed, China) according to the manufacturer’s instructions. Briefly, cell lysates were incubated with protein A + G beads and ZFP36L1 antibody or IgG overnight at 4 °C. The ZFP36L1-RNA complexes that had adsorbed to the beads were eluted, and the DNA was removed. RNA and the ZFP36L1 protein were separated from each other using a filter column. The enriched RNAs were analyzed by RT‒qPCR and electrophoresis.

### RNA pull-down assay

The PureBinding^TM^ RNA‒Protein pull-down Kit (Geneseed, China) was used to perform the RNA pull-down assay according to the manufacturer’s instructions. The SDC4 3’UTR, HIF1A 3’UTR, and VEGFA 3’UTR were synthesized by T7 RNA polymerase (Thermo Fisher Scientific, USA) and biotinylated with biotin RNA labeling mix (Sigma, USA) for use in the in vitro experiments. A total of 1 × 10^7^ cells were lysed with 1x capture buffer and incubated with magnetic beads and biotin-labeled probes for 1 h at 4 °C. After washing 3 times, the RNA-binding proteins were mixed with loading buffer (Beyotime, China) and analyzed by western blotting.

### Statistical analysis

All statistical analyses were performed using GraphPad Prism 8 software. The experimental results are presented as the means ± standard deviations (SD) of at least three independent experiments. The degree of variation within each dataset is depicted as SD in each figure. The groups exhibited similar variance. A two-tailed unpaired Student’s t test was used to determine statistical significance between two groups, and one-way or two-way ANOVA was used for multiple comparisons. Pearson’s χ^2^ test was performed to evaluate the correlation between ZFP36L1 expression levels and Vimentin or N-cadherin expression in OS patients. All statistical tests were justified as appropriate and the data met the assumptions of normal distribution. *p* < 0.05 was considered to indicate statistically significant differences (*P < 0.05; **P < 0.01; NS, not significant). Sample size was not predetermined using a specific statistical method.

### Supplementary information

Table. S1

Table. S2

Table. S3

Figure S1

Figure S2

Figure S3

Figure S4

Figure S5

Figure S6

Figure S7

Figure S8

Figure S9

Figure S10

Figure S11

Figure S12

Supplementary Figure legends

## Data Availability

The data generated in this study are available upon request from the corresponding author. Expression profile data analyzed in this study were obtained from Gene Expression Omnibus (GEO) at GSE18947, and GSE85537.
