# Supplementary material for: Low expression of ZFP36L1 in osteosarcoma promotes lung metastasis by inhibiting the SDC4-TGF-β signaling feedback loop
Source: Oncogene. 2023 Nov 7;43(1):47–60. doi: 10.1038/s41388-023-02880-7 (PMC10766520; doi:10.1038/s41388-023-02880-7)
Supplement: Supplementary file 1 — Table. S1 [file 41388_2023_2880_MOESM1_ESM.docx]

**Table. S1 Relationships between ZFP36L1 expression and the clinicopathological characteristics of 70 patients with osteosarcoma**

| **Characteristics** | **All patients** | **Expression of ZFP36L1** | | **p value** |
| --- | --- | --- | --- | --- |
|  |  | **Low (n=33)** | **High (n=37)** |  |
| **Sex** | P=0.2076 | | | |
| Male | 39 | 21 | 18 |  |
| Female | 31 | 12 | 19 |  |
| **Age (years)** | P=0.1057 | | | |
| ＜14 | 29 | 17 | 12 |  |
| ≥14 | 41 | 16 | 25 |  |
| **Anatomical site** | P=0.4483 | | | |
| Femur | 37 | 20 | 17 |  |
| Tibia | 17 | 7 | 10 |  |
| Humerus | 14 | 5 | 9 |  |
| Pelvis | 1 | 1 | 0 |  |
| Other | 1 | 0 | 1 |  |
| **Enneking’s stage** | P=0.0034 | | | |
| I A | 8 | 0 | 8 |  |
| I B | 13 | 3 | 10 |  |
| II A | 12 | 7 | 5 |  |
| II B | 20 | 12 | 8 |  |
| III | 17 | 12 | 5 |  |
