# Supplementary material for: Low expression of ZFP36L1 in osteosarcoma promotes lung metastasis by inhibiting the SDC4-TGF-β signaling feedback loop
Source: Oncogene. 2023 Nov 7;43(1):47–60. doi: 10.1038/s41388-023-02880-7 (PMC10766520; doi:10.1038/s41388-023-02880-7)
Supplement: Supplementary file 2 — Table. S2 [file 41388_2023_2880_MOESM2_ESM.docx]

**Table. S2 Primer sequences used for qRT-PCR**

| Genes | Primer |
| --- | --- |
| SAMD4A-F | CAGACCTCTCCTTGGGTGAC |
| SAMD4A-R | CACATTCGCAAACCCTCTGG |
| QKI-F | ATCATGGTCCGAGGCAAAGG |
| QKI-R | TGCAAACTTGTCTTGCCTCTCT |
| ZFP36L1-F | TCCAGCATAGCTTTAGCTTTGC |
| ZFP36L1-R | GGTCATCGGCGCTCAGAATAG |
| RBM26-F | GGAGGCAAATAGGTAGCCCTT |
| RBM26-R | GCGGATGGATCTGCATCACA |
| HNRNPA2B1-F | ATCTCTGCAAGGAGAAACACCT |
| HNRNPA2B1-R | TGATCACTTGGCTTGCCTCA |
| TRA2B-F | TCGATTGAAGCACATCGACC |
| TRA2B-R | TCTGGATCTAGACCCGCTCG |
| ZFR2-F | CAAGATGGCGACGAGTCAGT |
| ZFR2-R | CATCCCAGGAGTGGGTTGTG |
| CDH2-F | ACTTGCGAAACTCCAGACCC |
| CDH2-R | GCCGCTTTAAGGCCCTCATT |
| VIM-F | GGACCAGCTAACCAACGACA |
| VIM-R | AAGGTCAAGACGTGCCAGAG |
| SNAI1-F | CTCGGACCTTCTCCCGAATG |
| SNAI1-R | AAAGTCCTGTGGGGCTGATG |
| SDC4-F | GGACCTCCTAGAAGGCCGATA |
| SDC4-R | AGGGCCGATCATGGAGTCTT |
