# Supplementary material for: Low expression of ZFP36L1 in osteosarcoma promotes lung metastasis by inhibiting the SDC4-TGF-β signaling feedback loop
Source: Oncogene. 2023 Nov 7;43(1):47–60. doi: 10.1038/s41388-023-02880-7 (PMC10766520; doi:10.1038/s41388-023-02880-7)
Supplement: Supplementary file 3 — Table. S3 [file 41388_2023_2880_MOESM3_ESM.docx]

**Table. S3 Primer sequences used for ChIP assay**

| Genes | Primer |
| --- | --- |
| SBE1-F | TGGGGGTCTCATTATGGTTGC |
| SBE1-R | TCGGCTGGGCTCTATGGC |
| SBE2-F | GCCATAGAGCCCAGCCGA |
| SBE2-R | GGCCATACGATCCCTGTGG |
| SBE CTRL-F | CAACACCCTAGGGAAGTCCT |
| SBE CTRL-R | GAGATCACTGCGGCTAACCC |
| GAPDH-F | GAGCCTCGAGGAGAAGTTCC |
| GAPDH-R | GGACCCTTACACGCTTGGAT |
| SDC4-F | CCGAGTCGATGACTTGGAAGA |
| SDC4-R | ACTCATTGGTGGGGGCTTTC |
