# Supplementary figures and images for: Low expression of ZFP36L1 in osteosarcoma promotes lung metastasis by inhibiting the SDC4-TGF-β signaling feedback loop
Source: Oncogene. 2023 Nov 7;43(1):47–60. doi: 10.1038/s41388-023-02880-7 (PMC10766520; doi:10.1038/s41388-023-02880-7)

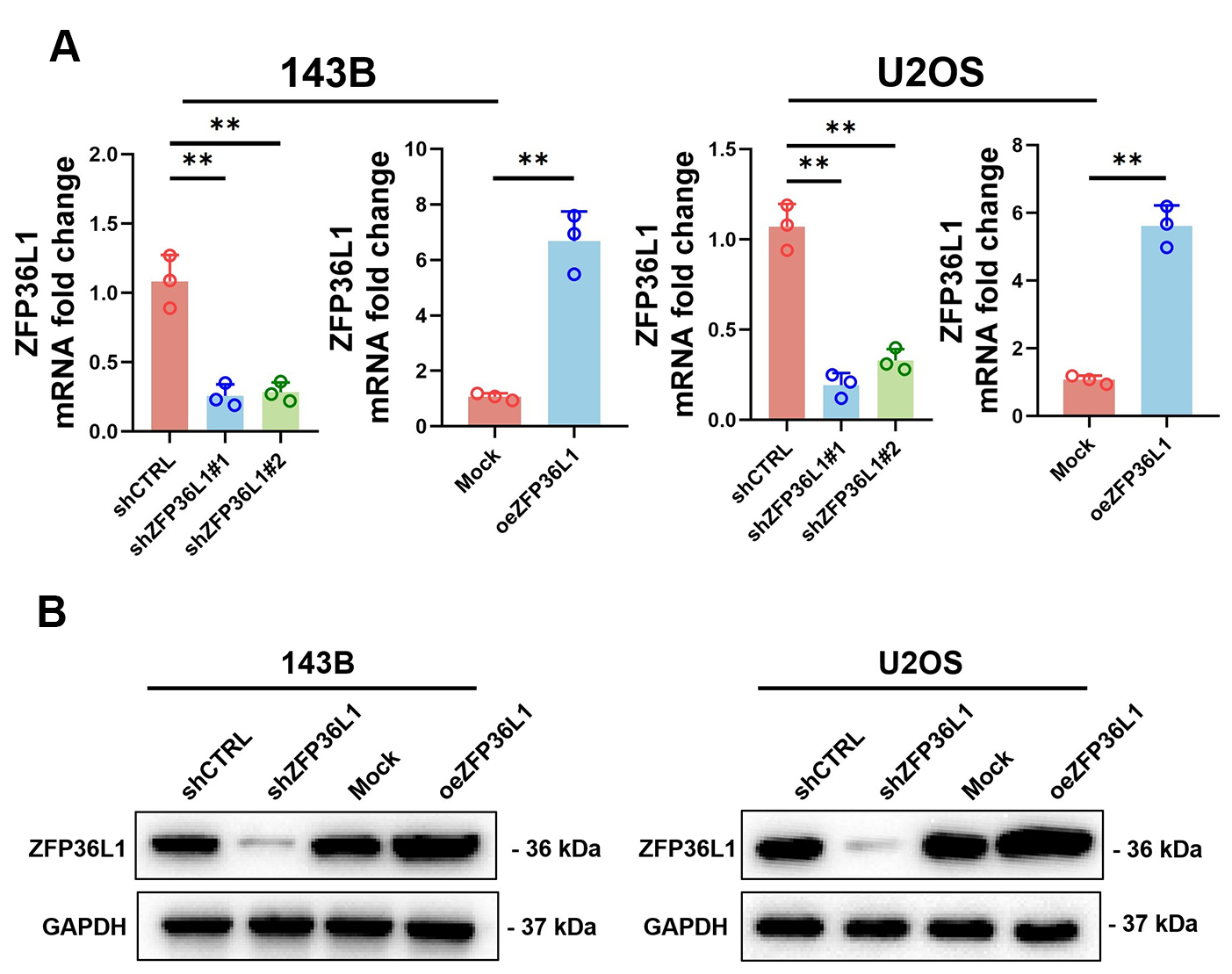

Supplement: Supplementary file 4 — Figure S1 [file 41388_2023_2880_MOESM4_ESM.png]

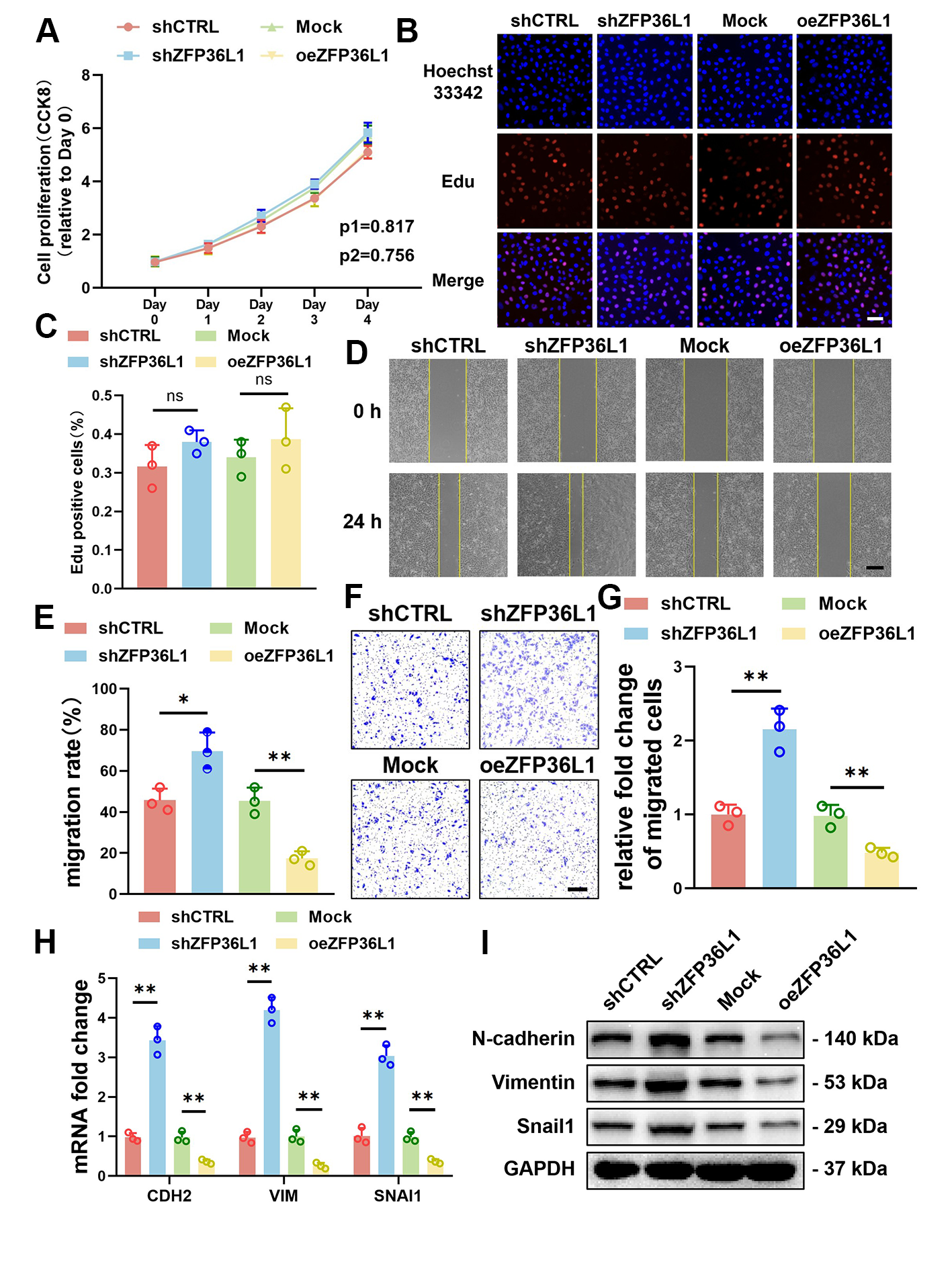

Supplement: Supplementary file 5 — Figure S2 [file 41388_2023_2880_MOESM5_ESM.png]

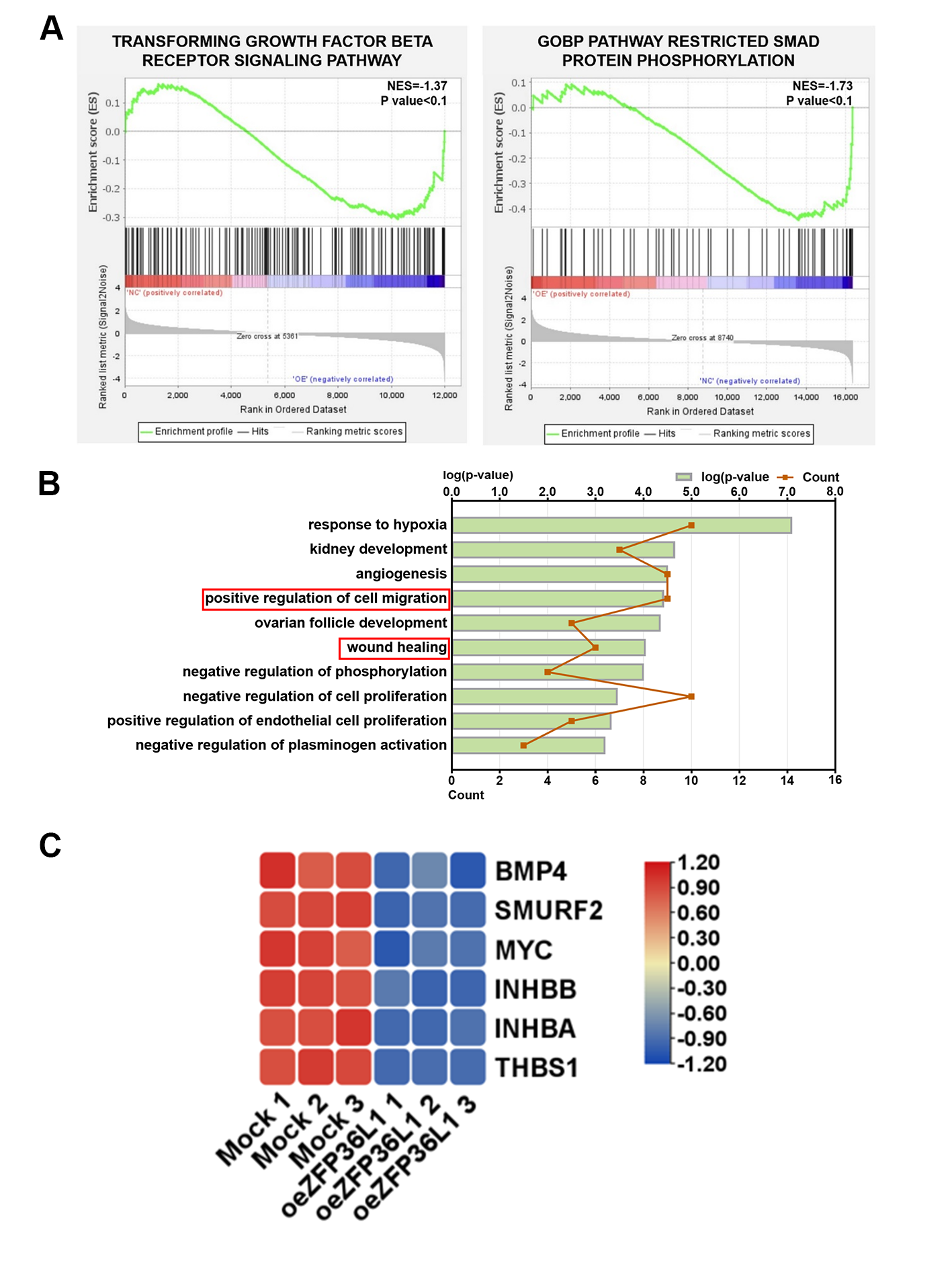

Supplement: Supplementary file 6 — Figure S3 [file 41388_2023_2880_MOESM6_ESM.png]

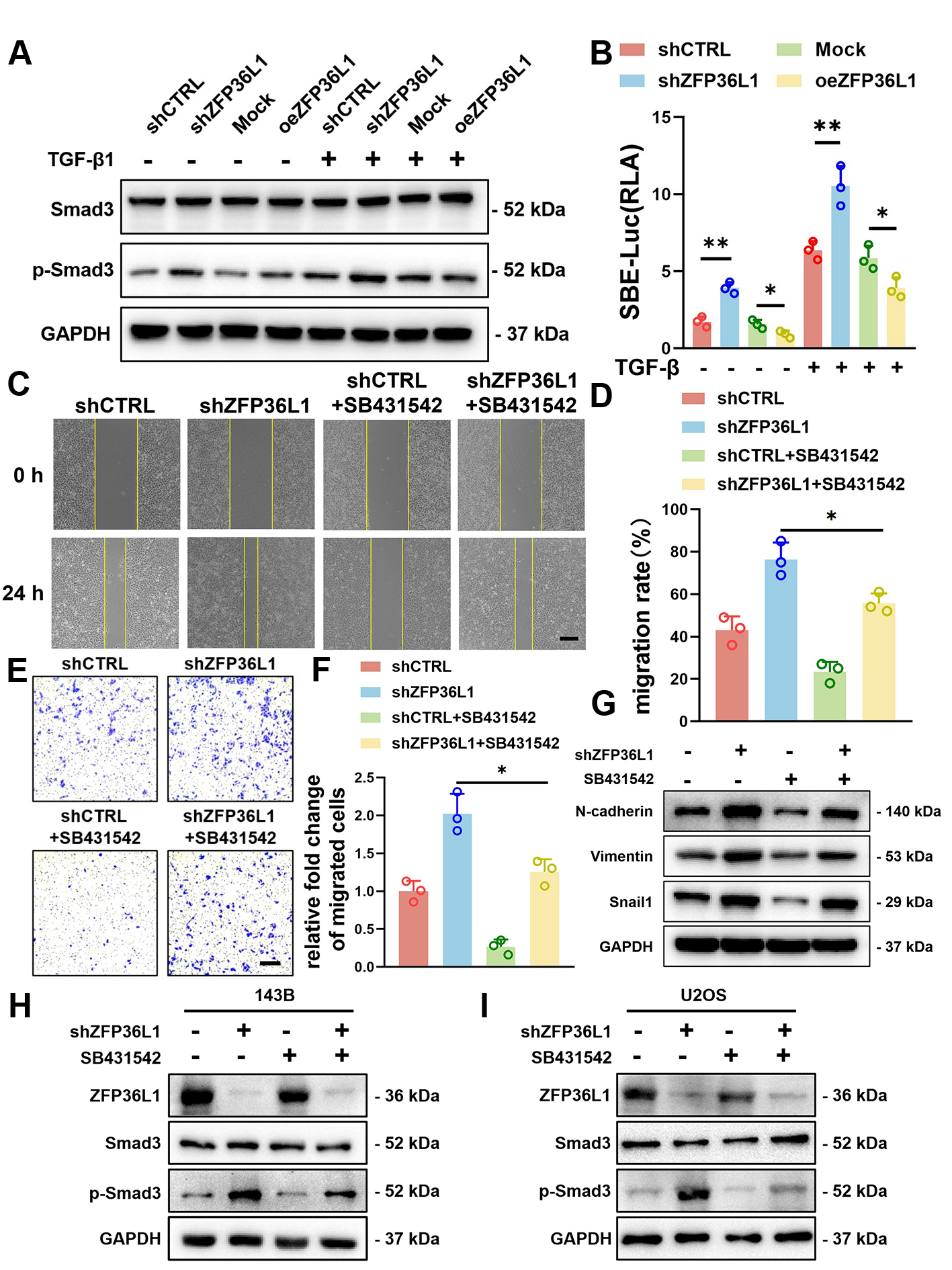

Supplement: Supplementary file 7 — Figure S4 [file 41388_2023_2880_MOESM7_ESM.png]

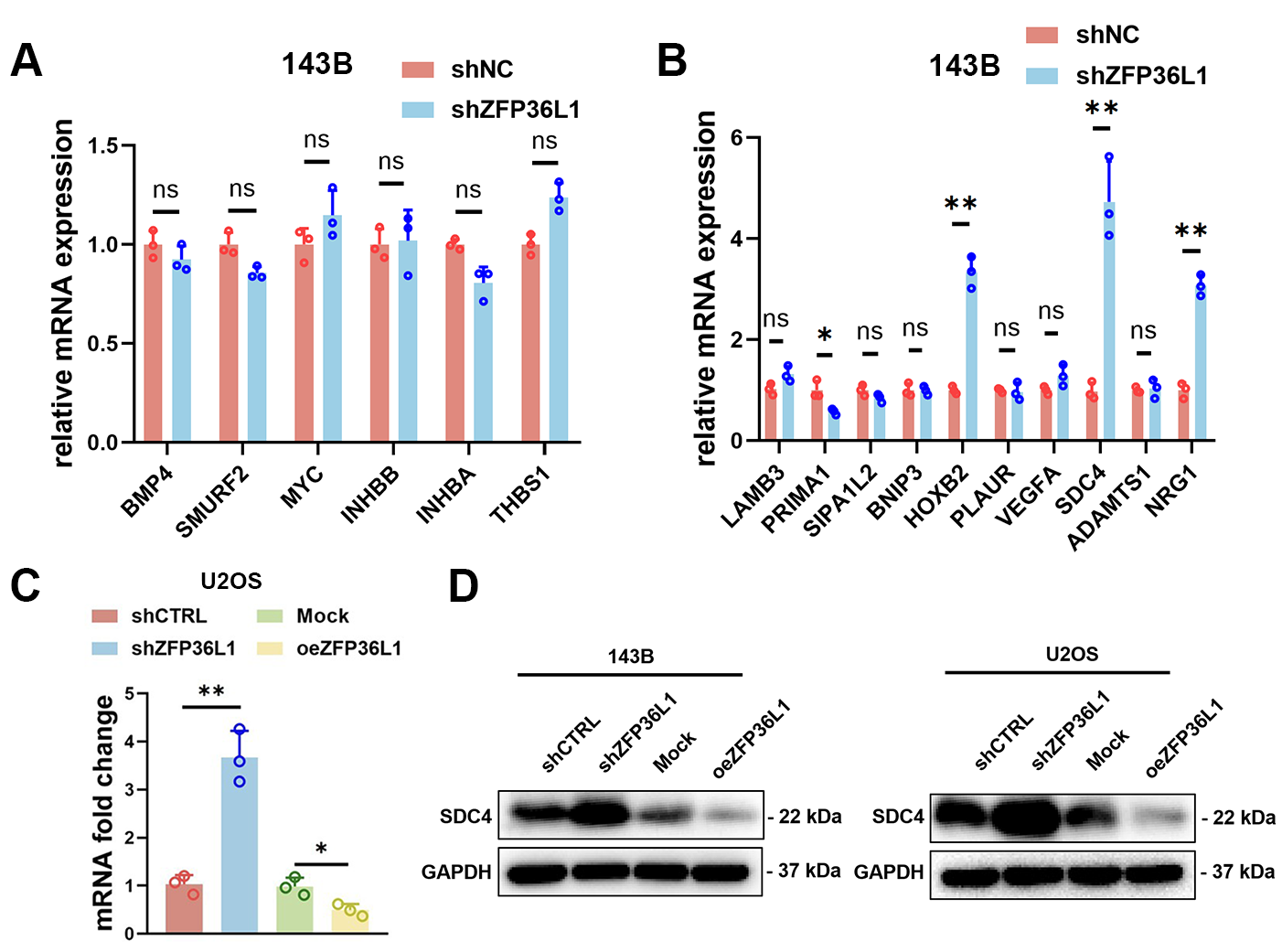

Supplement: Supplementary file 8 — Figure S5 [file 41388_2023_2880_MOESM8_ESM.png]

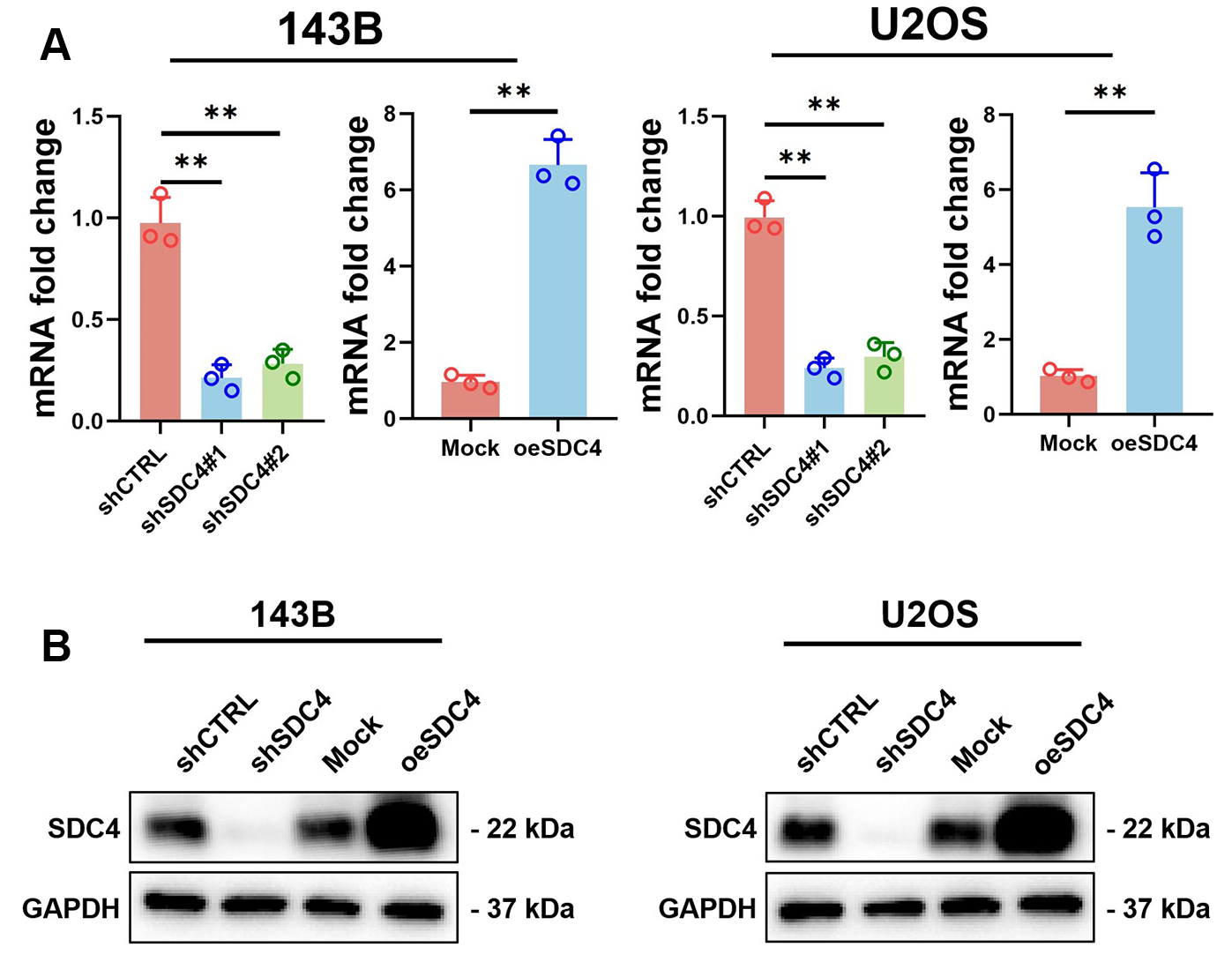

Supplement: Supplementary file 9 — Figure S6 [file 41388_2023_2880_MOESM9_ESM.png]

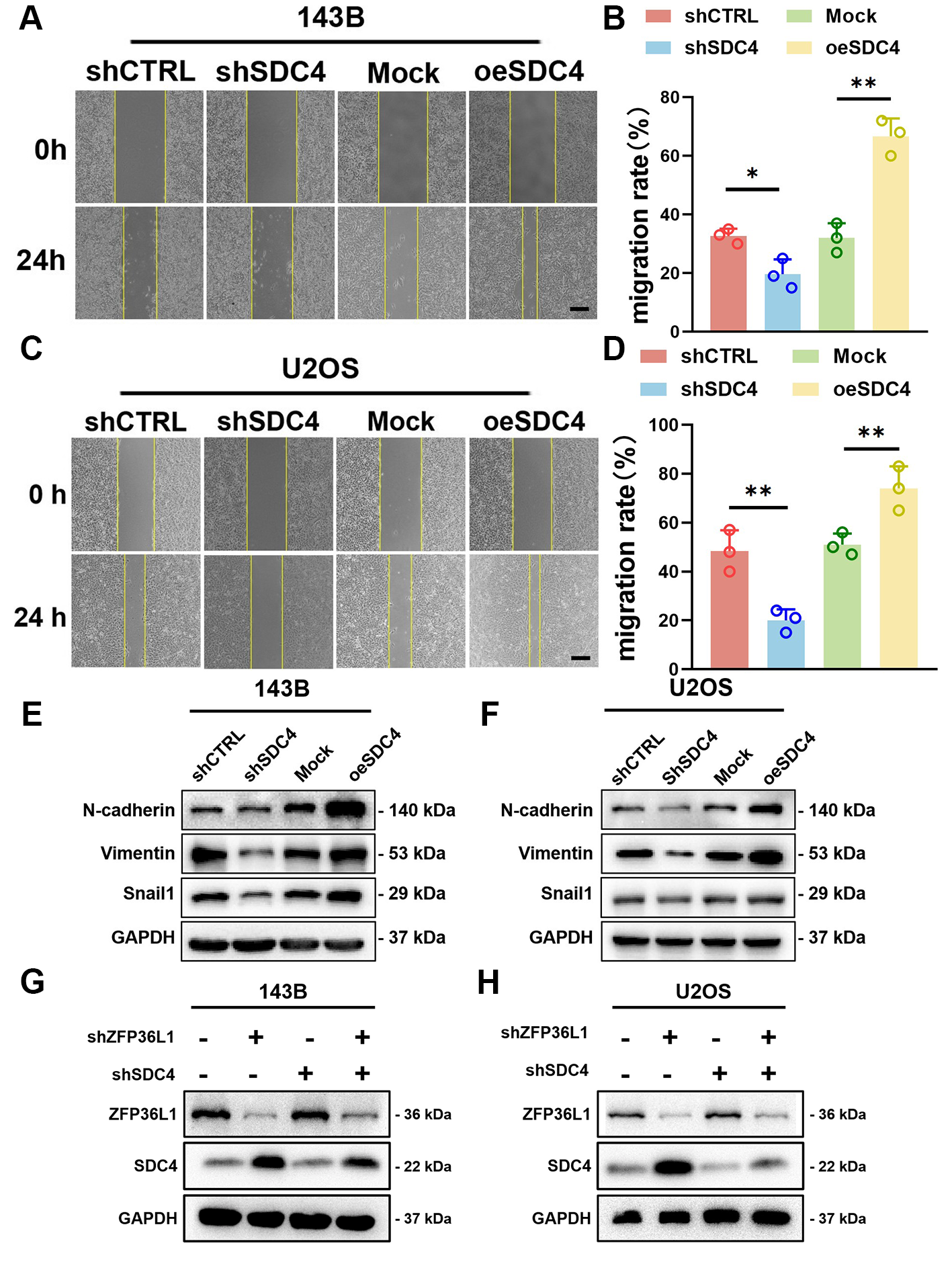

Supplement: Supplementary file 10 — Figure S7 [file 41388_2023_2880_MOESM10_ESM.png]

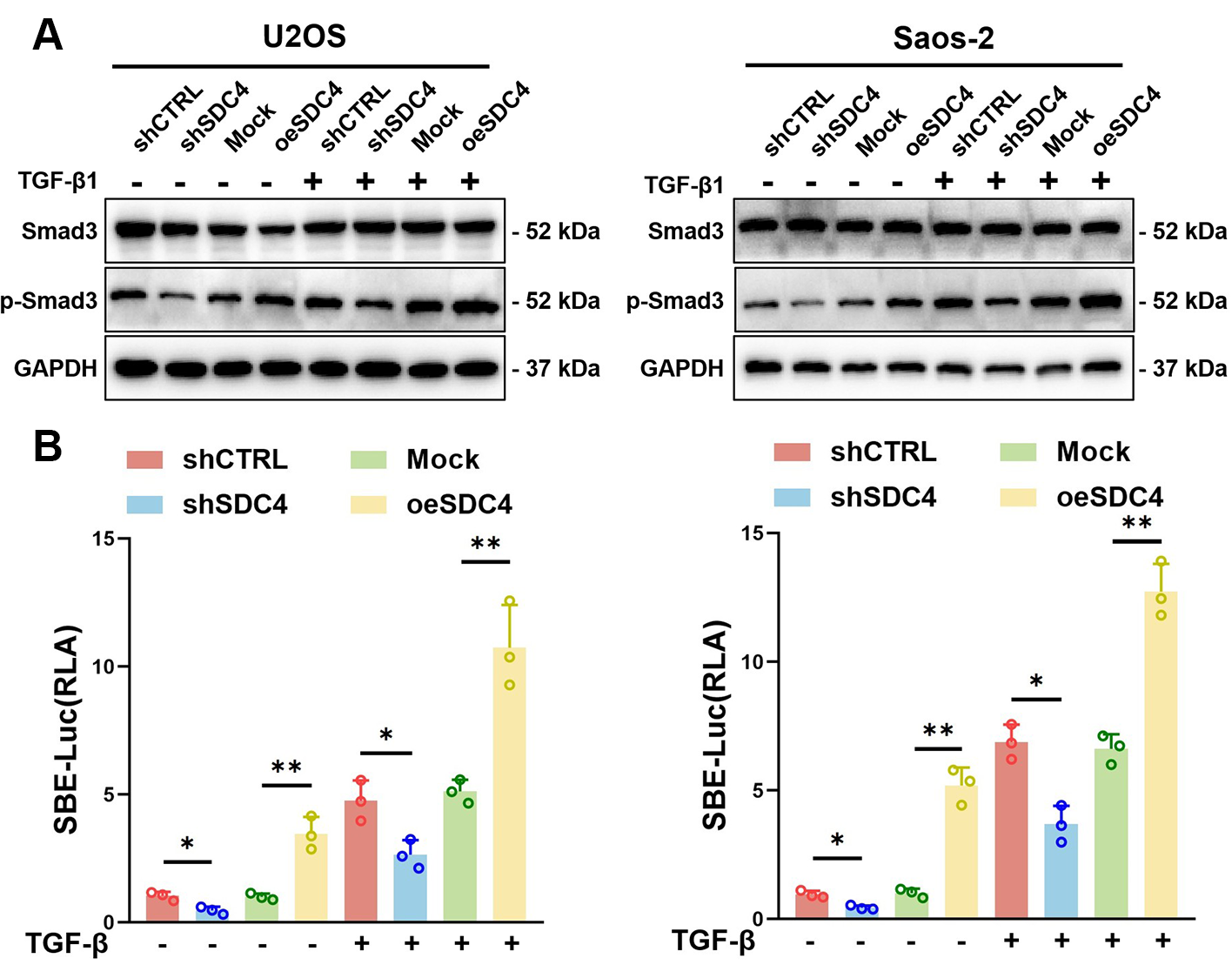

Supplement: Supplementary file 11 — Figure S8 [file 41388_2023_2880_MOESM11_ESM.png]

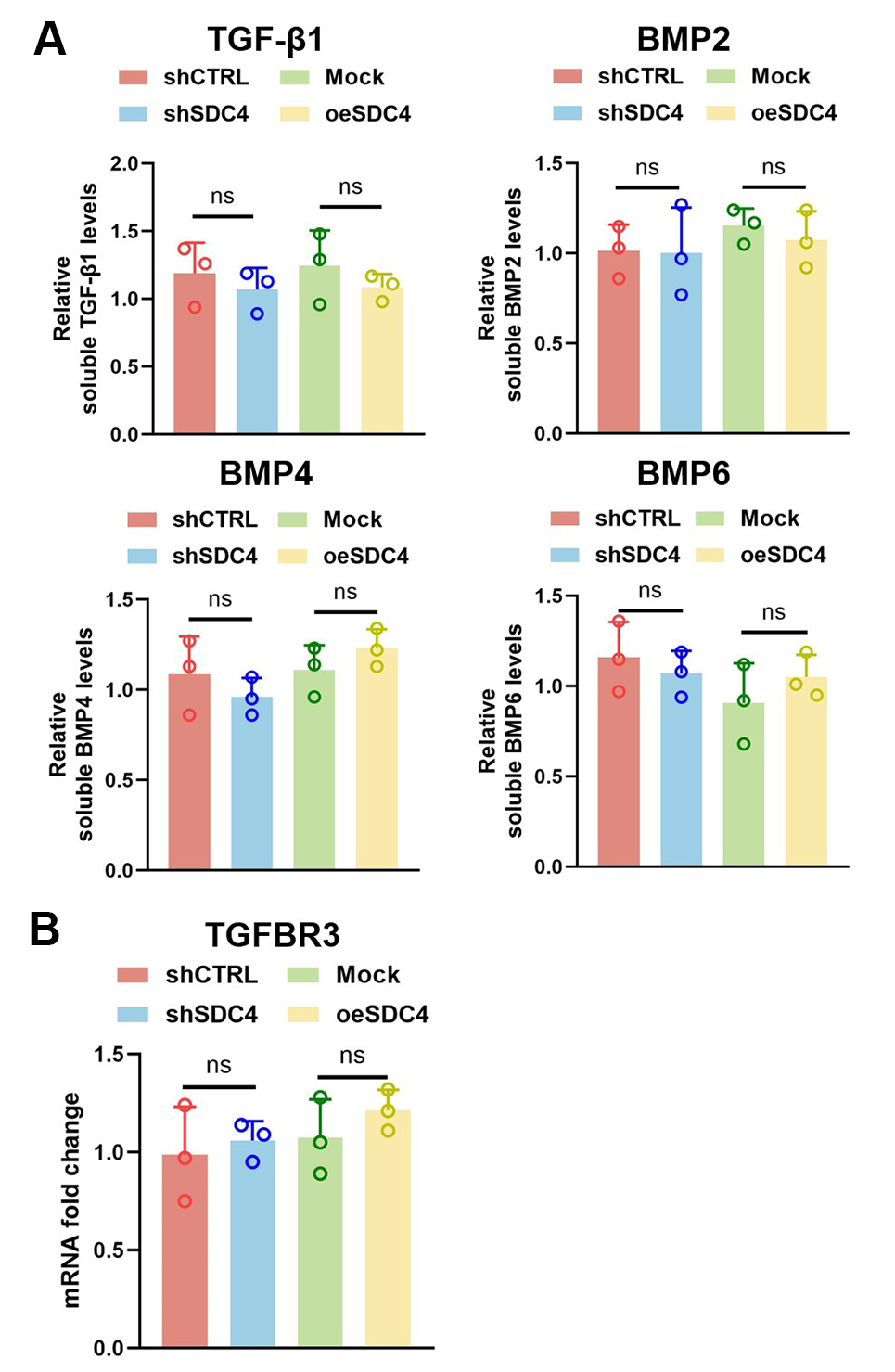

Supplement: Supplementary file 12 — Figure S9 [file 41388_2023_2880_MOESM12_ESM.png]

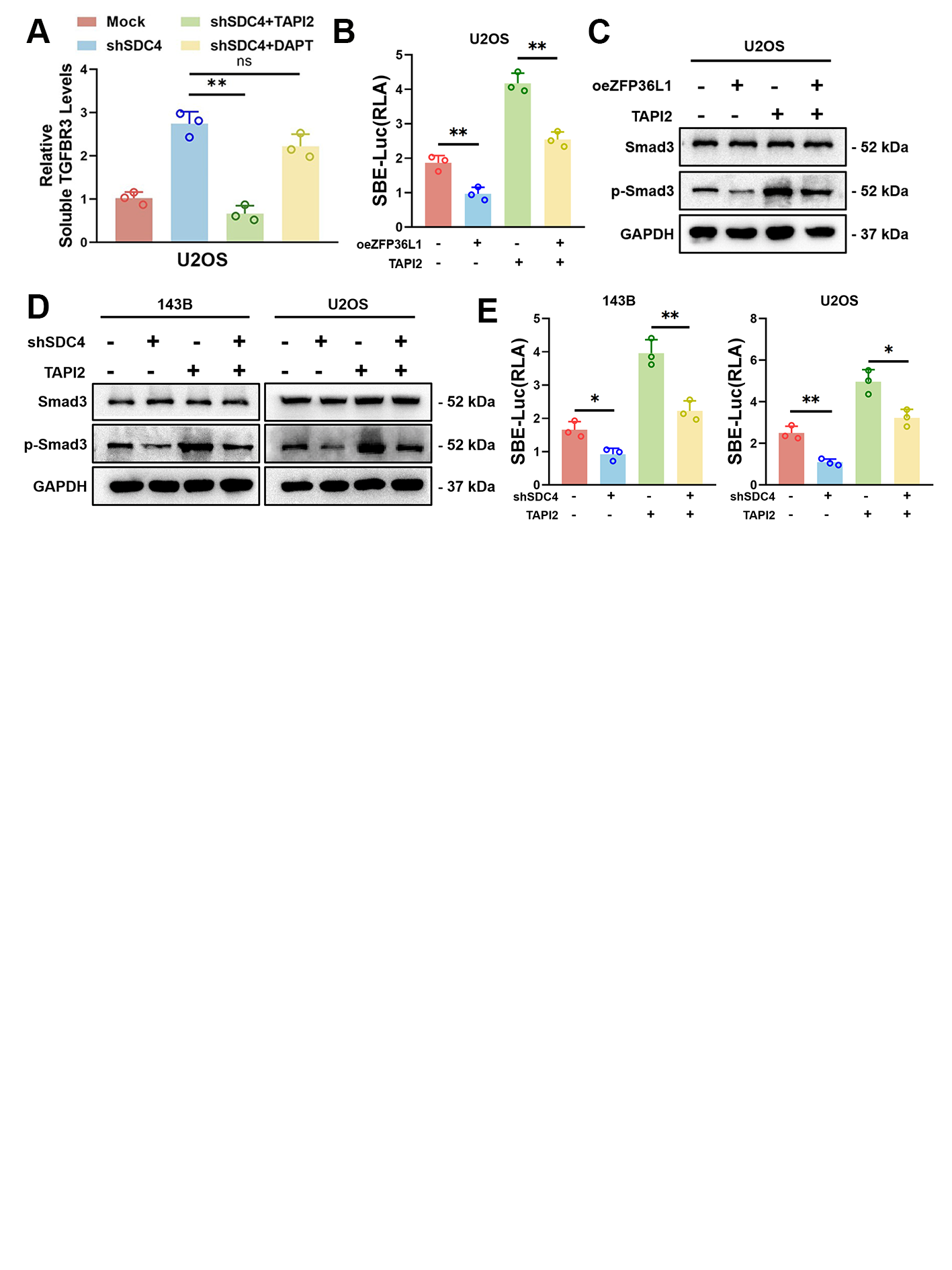

Supplement: Supplementary file 13 — Figure S10 [file 41388_2023_2880_MOESM13_ESM.png]

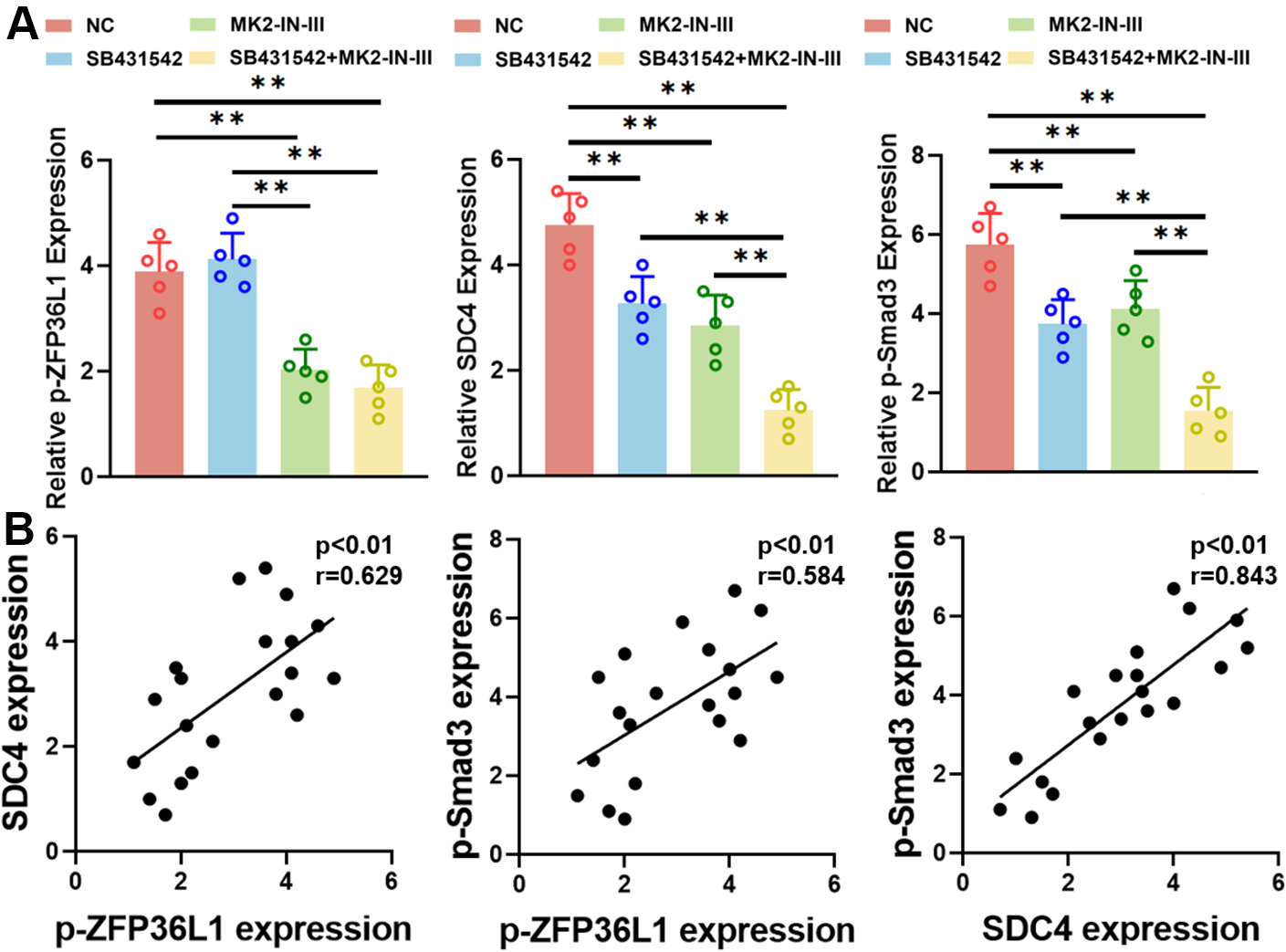

Supplement: Supplementary file 14 — Figure S11 [file 41388_2023_2880_MOESM14_ESM.png]

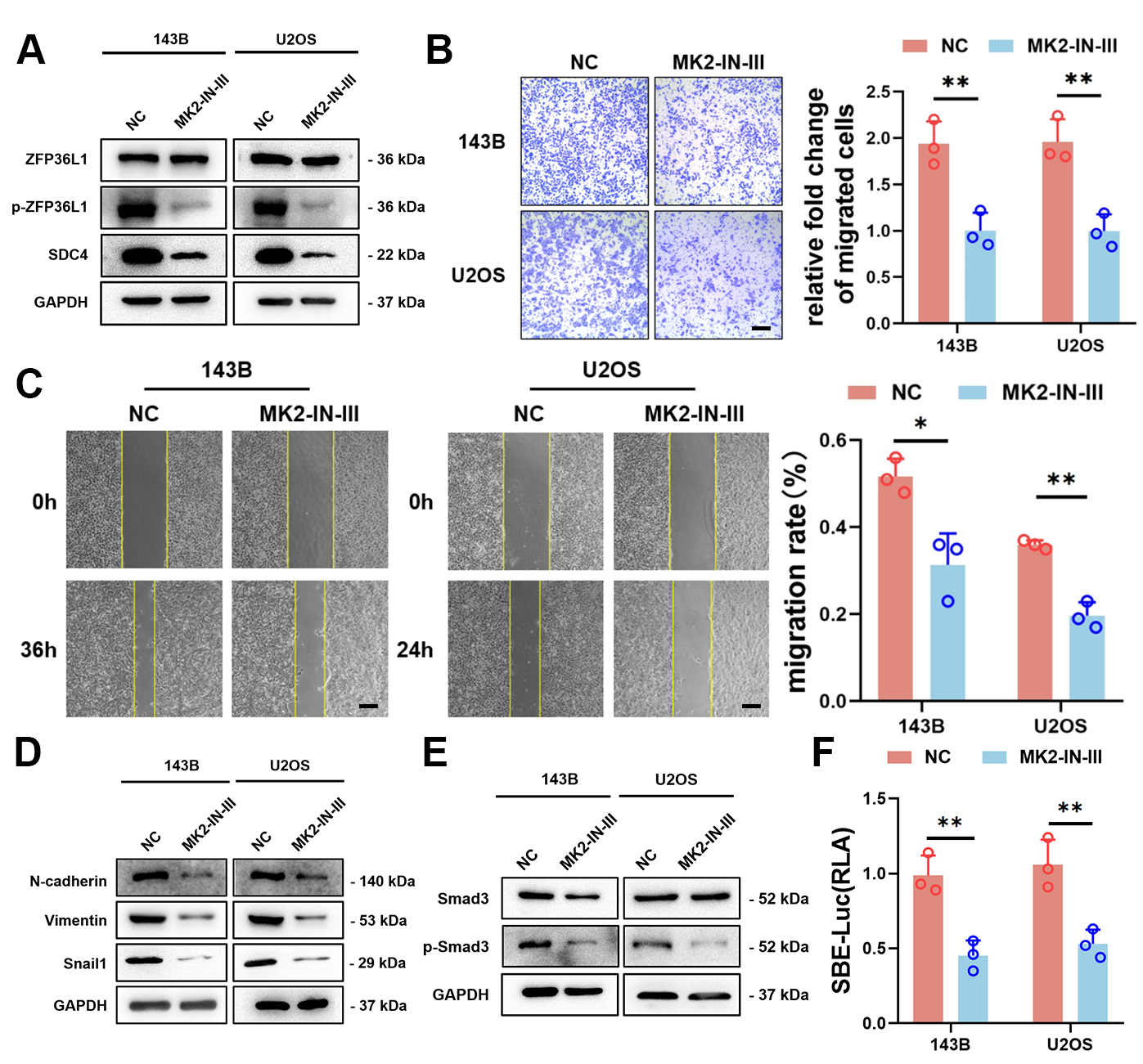

Supplement: Supplementary file 15 — Figure S12 [file 41388_2023_2880_MOESM15_ESM.png]
