# Supplementary material for: Low expression of ZFP36L1 in osteosarcoma promotes lung metastasis by inhibiting the SDC4-TGF-β signaling feedback loop
Source: Oncogene. 2023 Nov 7;43(1):47–60. doi: 10.1038/s41388-023-02880-7 (PMC10766520; doi:10.1038/s41388-023-02880-7)
Supplement: Supplementary file 16 — Supplementary Figure legends [file 41388_2023_2880_MOESM16_ESM.docx]

**Supplementary Figure legends**

**Fig. S1 ZFP36L1 overexpression and knockdown cell lines were constructed.** **A-B**, q-PCR assays **(A)** and WB **(B)** confirmed ZFP36L1 overexpression and knockdown in 143B cells and U2OS cells. The data are shown as means ± SEMs; *P < 0.05, **P < 0.01.

**Fig. S2 Effect of ZFP36L1 on the proliferation, migration and EMT of U2OS cells. A-C**, CCK8 assays **(A)** and EdU staining assays **(B)** were conducted to determine the proliferation rate of oeZFP36L1 or shZFP36L1 U2OS cells. The percentage of EdU-positive cells **(C)** was determined by ImageJ. Scale bar, 80 μm. **D-G**, The migration of U2OS cells was measured using wound healing tests **(D)** and Transwell migration assays **(F)** and quantitatively analyzed **(E and G)** with ImageJ. Scale bar, 150 μm. **H** and **I**, q-PCR assays **(H)** and WB **(I)** were performed to examine the role of ZFP36L1 in regulating EMT-associated gene expression in U2OS cells. The data are shown as means ± SEMs; *P < 0.05, **P < 0.01.

**Fig. S3** **Bioinformatics analysis of RNA-seq results. A**, The gene expression profiles of both mock and oeZFP36L1 143B cells were subjected to Gene Set Enrichment Analysis (GSEA) within two gene sets associated with the TGF-beta pathway. **B**, Gene Ontology Biological Process (GOBP) analysis conducted on the differentially expressed genes (log_2_(oeZFP36L1/mock)≥1) identified through transcriptome sequencing of both mock and oeZFP36L1 143B cells. **C**, Heatmap of differentially expressed genes associated with the TGF-β signaling pathway in RNA-seq.

**Fig. S4 ZFP36L1 inhibited U2OS cell migration by regulating the TGF-β signaling pathway.** **A-B**, WB **(A)** and SBE-luciferase activity **(B)** assays were performed to determine the role of ZFP36L1 in the TGF-β signaling pathway in U2OS cells. **C-F**, The migration of shZFP36L1 U2OS cells stimulated with or without SB431542 was measured by wound healing assays **(C)** and Transwell assays **(E)** and quantitatively analyzed with ImageJ **(D and F)**. Scale bar, 150 μm. **G**, The impact of SB431542 on ZFP36L1-mediated regulation of EMT-associated gene expression in U2OS cells was determined by WB. **H-I**, WB was conducted to examine the effect of shZFP36L1 and SB431542 on modulating TGF-β pathway activity in 143B cells or U2OS cells. The data are shown as means ± SEMs; *P < 0.05, **P < 0.01.

**Fig. S5 Effect of ZFP36L1 on SDC4 expression in OS cells. A**, q-PCR assays were performed to investigate the influence of shZFP36L1 on TGF-β signaling pathway associated gene expression in 143B cells. These genes were identified in KEGG analysis of RNA-seq data. **B**, q-PCR assays were conducted to determine the impact of shZFP36L1 on gene expression in 143B cells. These genes were selected based on their ranking in the top 10 genes with the highest p-values among genes with log_2_(oeZFP36L1/mock)≥1, as identified in our RNA-seq data. **C-D**, q-PCR assays **(C)** and WB **(D)** were performed to confirm the effect of ZFP36L1 on SDC4 expression in OS cells. The data are shown as means ± SEMs; *P < 0.05, **P < 0.01.

**Fig. S6. SDC4 overexpression and knockdown cell lines were constructed. A-B**, q-PCR assays **(A)** and WB **(B)** confirmed SDC4 overexpression and knockdown in 143B cells and U2OS cells, respectively. The data are shown as means ± SEMs; *P < 0.05, **P < 0.01.

**Fig. S7 Effect of SDC4 on OS cell migration and EMT. A-D**, Migration in 143B cells **(A)** and U2OS cells **(C)** was measured by wound healing assays and quantitatively analyzed **(B and D)** with ImageJ. Scale bar, 150 μm. **E-F**, The impact of SDC4 on regulating EMT-associated gene expression in 143B cells **(E)** and U2OS cells **(F)** was examined by WB. **G-H**, WB were performed to assess SDC4 expression under condition of shZFP36L1 or shSDC4 treatment. The data are shown as means ± SEMs; *P < 0.05, **P < 0.01.

**Fig. S8** **Effect of SDC4 on TGF-β signaling pathway activation in OS cells. A-B**, WB **(A)** and SBE-luciferase activity **(B)** assays were performed to determine the role of SDC4 in the TGF-β signaling pathway in U2OS and Saos-2 cells. The data are shown as means ± SEMs; *P < 0.05, **P < 0.01.

**Fig. S9 Effect of SDC4 on the expression of TGF-β family members. A**, ELISA was performed to analyze the impact of SDC4 on regulating the secretion of TGF-β family members (TGF-β1, BMP2, BMP4 and BMP6) by 143B cells. **B**, q-PCR assays were conducted to confirm the impact of SDC4 on TGFBR3 expression. The data are shown as means ± SEMs; *P < 0.05, **P < 0.01.

**Fig. S10 Impact of TAPI2 on the attenuation of TGF-β pathway activity mediated by oeZFP36L1 or shSDC4 in osteosarcoma cells. A,** ELISA was conducted to measure TGFBR3 cleavage regulated by TAPI2 or DAPT in U2OS cells. **B-C**, WB **(B)** and SBE-luciferase activity **(C)** assays were conducted to elucidate the role of TAPI2 in the modulation of the TGF-β signaling pathway attenuated by oeZFP36L in U2OS cells. **D-E**, WB **(D)** and SBE-luciferase activity **(E)** assays were performed to determine the role of TAPI2 in the inhibition of the TGF-β signaling pathway by shSDC4 in 143B and U2OS cells. The data are shown as means ± SEMs; *P < 0.05, **P < 0.01.

**Fig. S11 Quantitative Analysis of IHC Staining in Osteosarcoma Cells within Mouse Lung Tissue. A,** Relative expression levels of p-ZFP36L1, SDC4, and p-Smad3 in distinct groups of osteosarcoma cells within mouse lung tissue as determined by IHC staining. **B,** Linear correlations between p-ZFP36L1 and SDC4, p-ZFP36L1 and p-Smad3, and SDC4 and p-Smad3 according to IHC staining of osteosarcoma cells within mouse lung tissue. The data are shown as means ± SEMs; *P < 0.05, **P < 0.01.

**Fig. S12 The role of MK2-IN-III in enhancing ZFP36L1-mediated suppression of migration and EMT in osteosarcoma cells. A,** WB was performed to elucidate the role of MK2-IN-III in inhibiting the phosphorylation of ZFP36L1 and SDC4 expression. **B-C**, The effect of MK2-IN-III on the migration ability of OS cells was examined by Transwell **(B)** and wound healing **(C)** assays. Scale bar, 150 μm. **D**, WB was conducted to delineate the involvement of TAPI2 in regulating EMT-associated gene expression in OS cells. **E-F**, WB and SBE-luciferase activity assays were carried out to indicate the impact of MK2-IN-III on regulating the TGF-β signaling pathway in OS cells. The data are shown as means ± SEMss; *P < 0.05, **P < 0.01.
